# Supplementary material for: Worldwide divergence of values
Source: Nat Commun. 2024 Apr 9;15:2650. doi: 10.1038/s41467-024-46581-5 (PMC11004123; doi:10.1038/s41467-024-46581-5)
Supplement: Supplementary file 1 — Supplementary Information [file 41467_2024_46581_MOESM1_ESM.pdf]

SUPPLEMENTARY FIGURES **2**

Supplementary Figure 1. .... *2*
Supplementary Figure 2. .... *3*
Supplementary Figure 3. .... *4*
Supplementary Figure 4. .... *5*
Supplementary Figure 5. .... *6*
Supplementary Figure 6. .... *7*
Supplementary Figure 7. .... *8*

SUPPLEMENTARY TABLES **9**

Supplementary Table 1. .... *9*
Supplementary Table 2. .... *13*
Supplementary Table 3. .... *15*
Supplementary Table 4. .... *17*
Supplementary Table 5. .... *19*
Supplementary Table 6. .... *21*
Supplementary Table 7. .... *22*
Supplementary Table 8. .... *23*
Supplementary Table 9. .... *24*
Supplementary Table 10. .... *25*
Supplementary Table 11. .... *26*
Supplementary Table 12. .... *27*
Supplementary Table 13. .... *28*
Supplementary Table 14. .... *29*
Supplementary Table 15. .... *30*
Supplementary Table 16. .... *31*
Supplementary Table 17. .... *32*

SUPPLEMENTARY METHODS **33**

EXTENDED MATERIALS AND METHODS 33
The World Values Survey. *33*
Exogenous Variables. *34*
ANALYTIC STRATEGY 36
Item Normalization. *36*
Calculating Value Divergence Across Items. *36*
Calculating Value Distinctiveness. *37*
Clustering Methodology. *38*
SUPPLEMENTAL LITERATURE REVIEW 38
ADDITIONAL ANALYSES 40
Replicating Results Without Demographic Weighting. *40*
Replicating Results with Median Split Item Normalization. *40*
Replicating Results Controlling for Geographic Distinctiveness. *41*
Replicating Results with Cultural Fixation Indices. *42*
Replicating Analyses of Value Distinctiveness Without GDP Per Capita. *43*
Separating Longitudinal and Cohort Effects with Centering. *43*
Non-Linear Value Divergence. *43*
Within-Country Heterogeneity Analyses. *44*
Wealth and Value Distinctiveness by Continent. *44*
Correlating PCs with Dimensions of Values. *45*
Visualizing Country Clusters at Each Timepoint. *45*
Additional Examples of Value Divergence. *46*
Full Statistics for Clustering Regressions. *46*

SUPPLEMENTARY REFERENCES **47**

Supplementary Figures

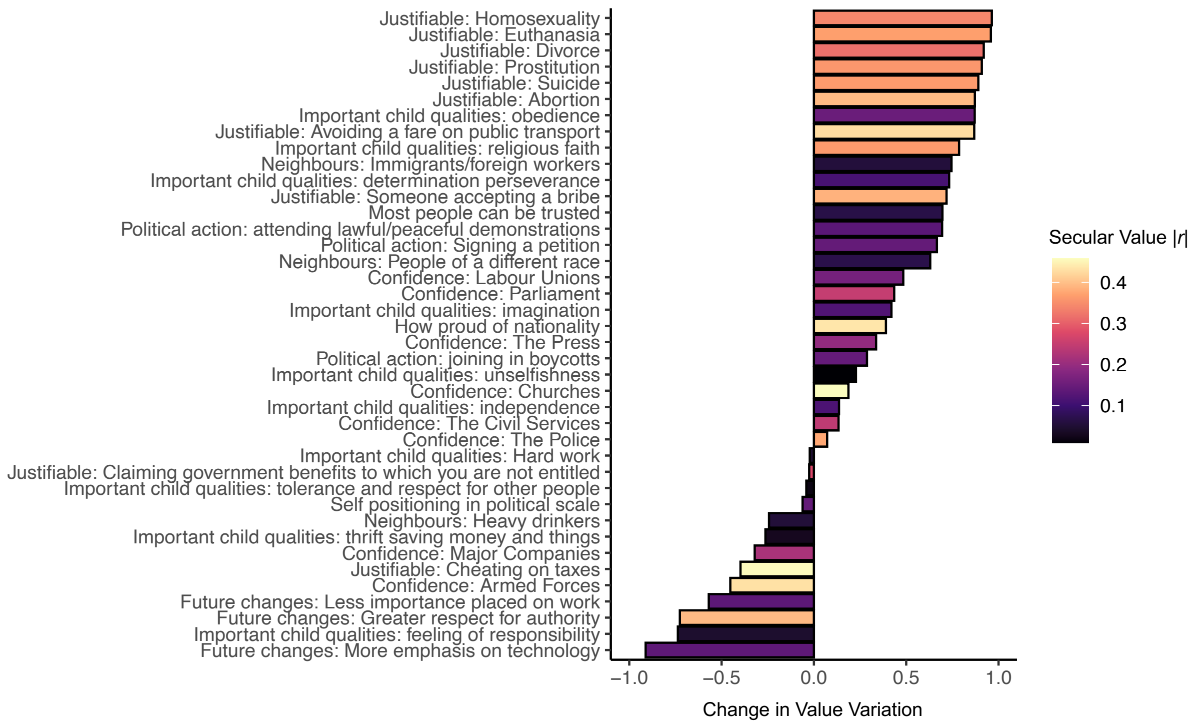

Supplementary Figure 1. Value divergence across all 40 items in our analysis, as indexed by the correlation between time and value variation for each item (using a median split normalization approach). The y-axis provides each item label associated with the longitudinal WVS dataframe. The fill color indicates the absolute value of the correlation of each item with Welzel's index of secular vs. sacred values[8]; brighter bars correlate either highly positively or negatively on this index.

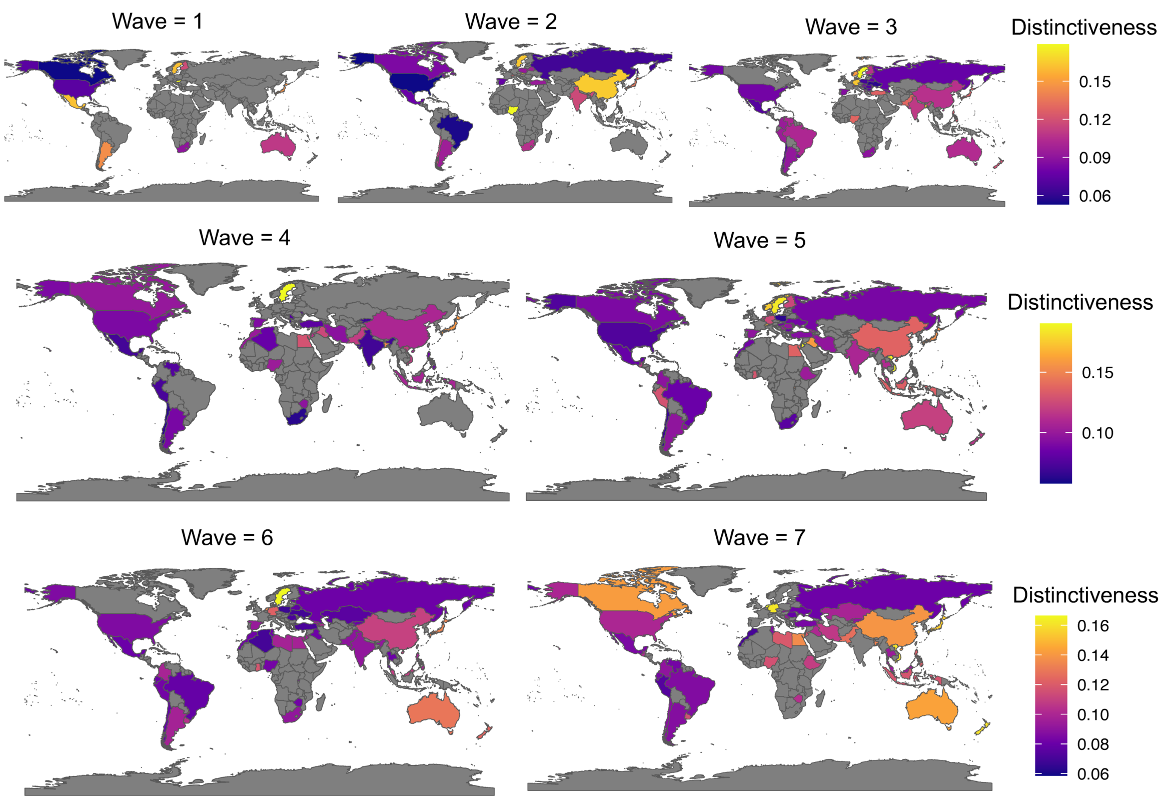

Supplementary Figure 2. World maps displaying value distinctiveness for each WVS wave. Brighter colors represent more value distinctiveness, meaning that countries had more unique values, on average, than other countries included in the sample.

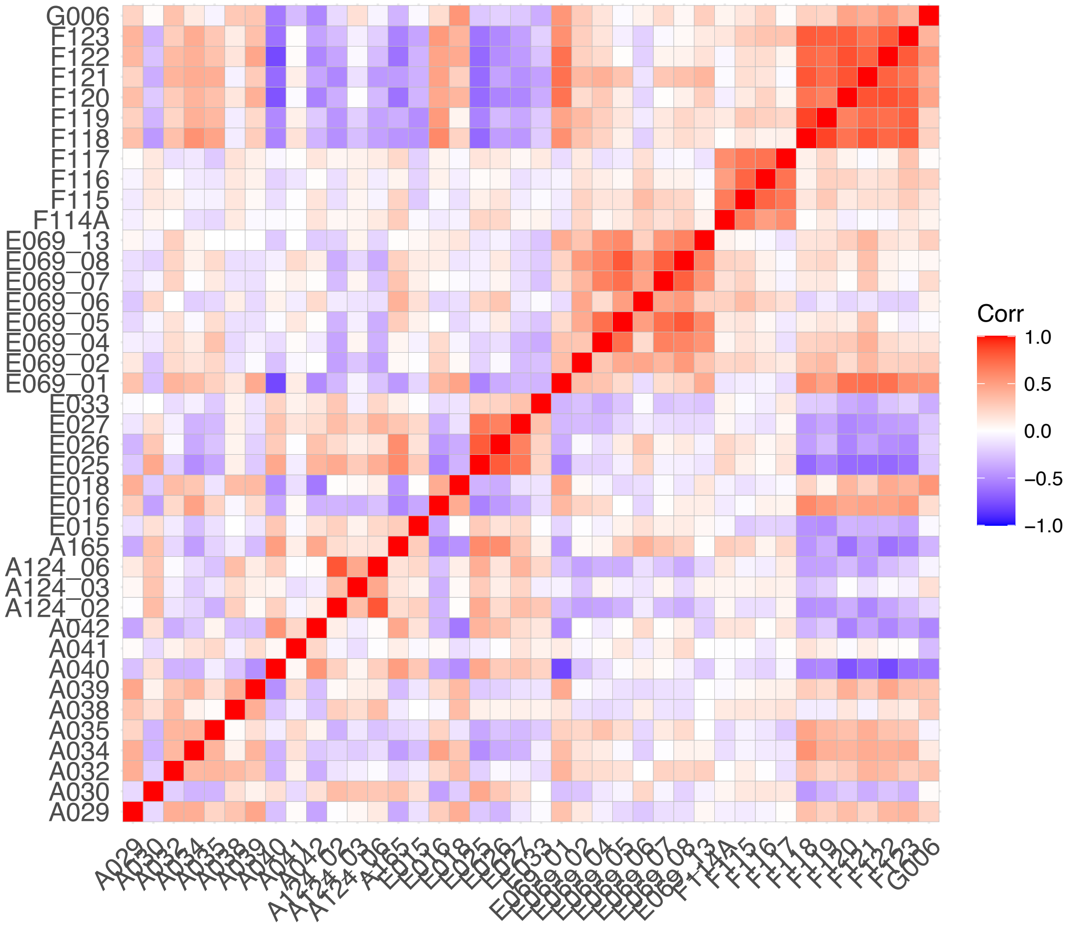

Supplementary Figure 3. Pearson correlations of value means across the 40 values that comprised our analyses. Redder squares represent more positive correlations, and bluer squares represent more negative correlations.

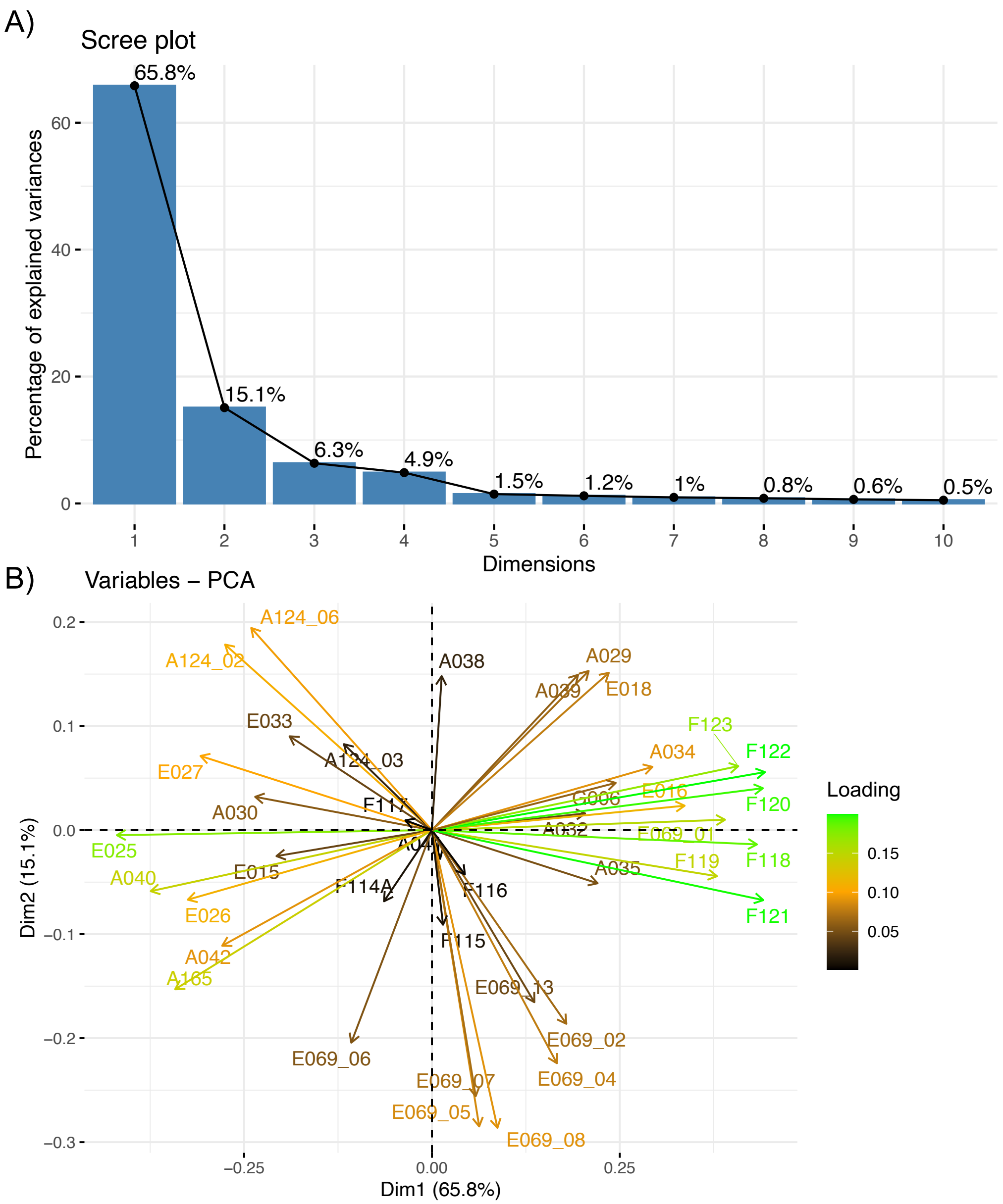

Supplementary Figure 4. Panel A) An elbow plot of variance explained by each PC in our PCA. Panel B) The loadings of each item (by ID) on PC1 and PC2.

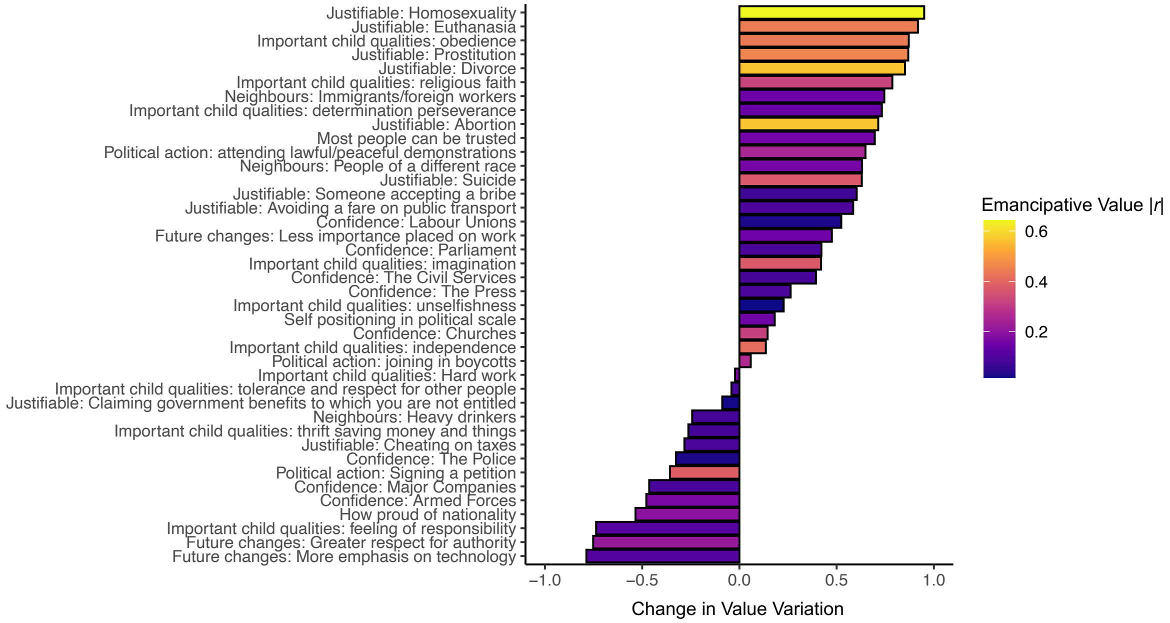

Supplementary Figure 5. Value divergence across all 40 items in our analysis, as indexed by the correlation between time and value variation for each item (using a median split normalization approach). The y-axis provides each item label associated with the longitudinal WVS dataframe. The fill color indicates the absolute value of the correlation of each item with Welzel's index of emancipative values[8]; brighter bars correlate either highly positively or negatively on this index.

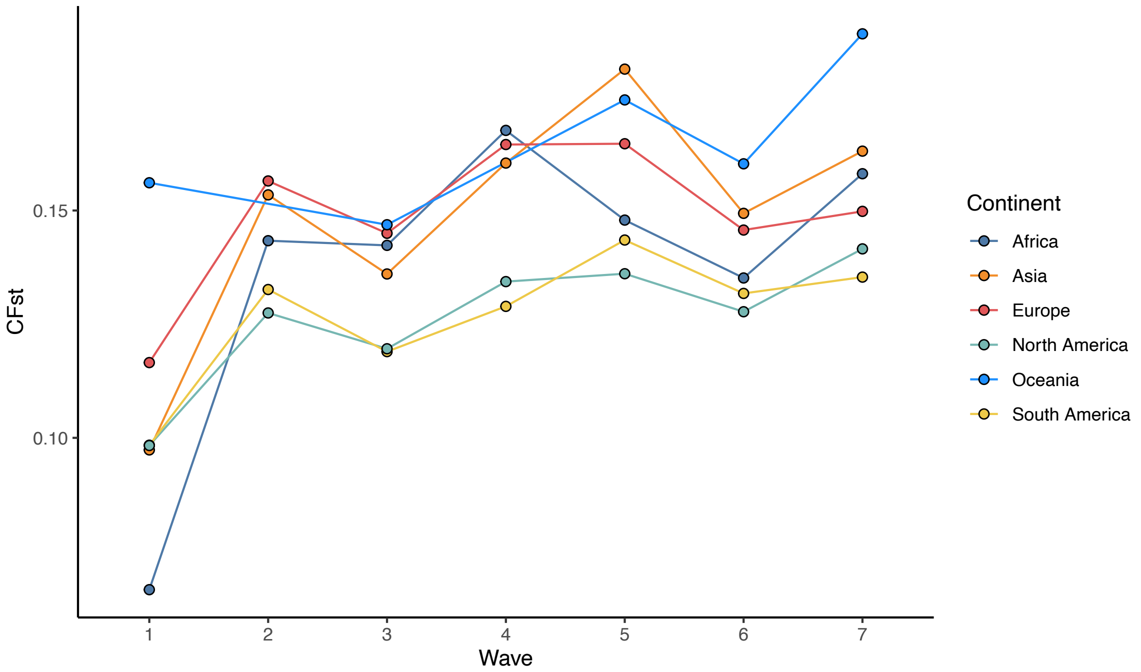

Supplementary Figure 6. Continents by their mean CF_{ST} score (contrasted with countries in other continents) at each WVS wave. Mean CF_{ST} has risen for all continents over time, which is consistent with value divergence.

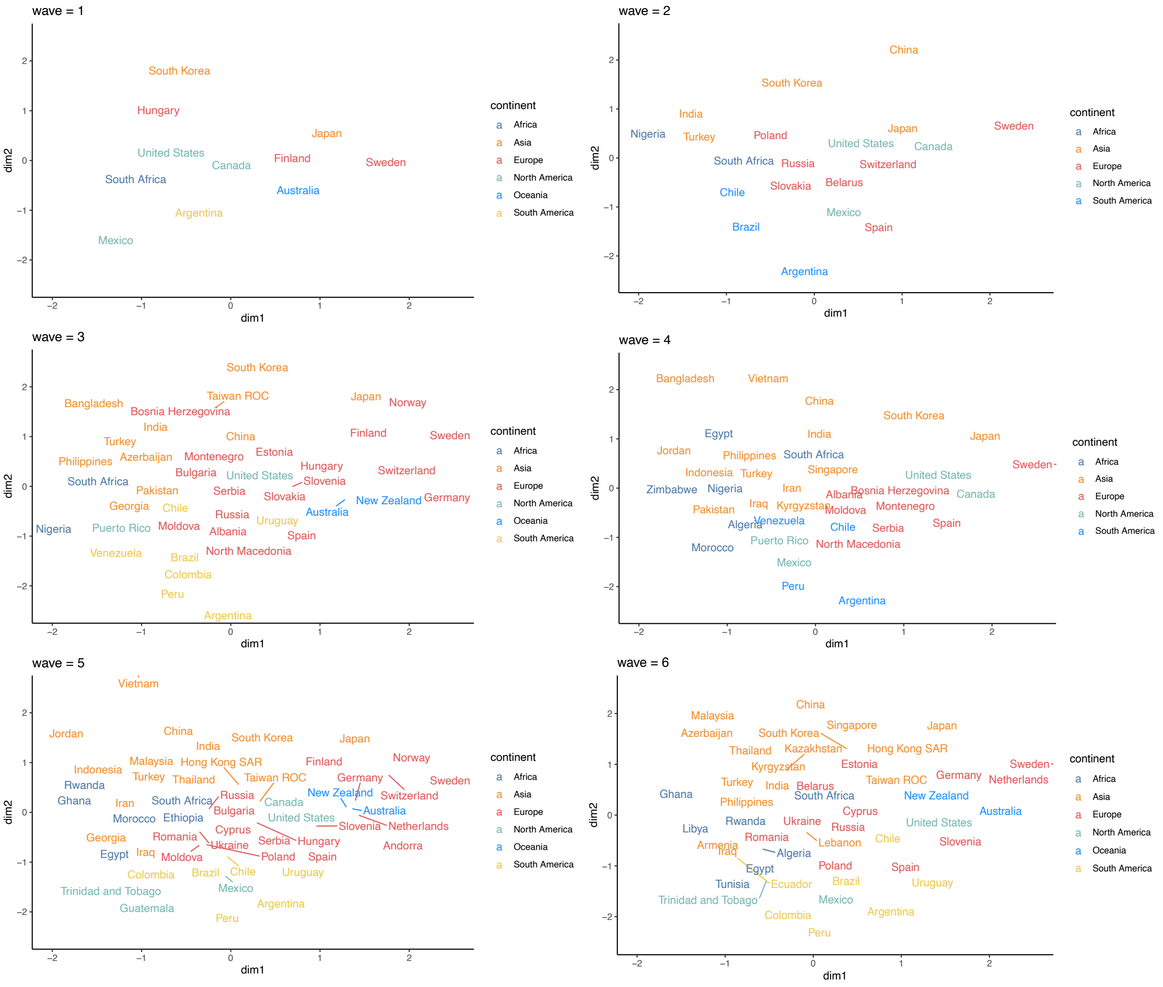

Supplementary Figure 7. Wave 1-6 countries projected on a two-dimensional value space derived from a PCA of all 40 values in our analysis. Proximity in this space indicates that two countries have similar values.

Supplementary Tables

Supplementary Table 1.
Demographic Characteristics of Countries by WVS Wave

**Country**	**Variable**	**Wave**						
		1	2	3	4	5	6	7
Argentina	% Female	0.51	0.53	0.53	0.53	0.53	0.53	0.52
	Mean Age	41.59	42.89	42.73	42.05	42.55	43.17	42.55
Australia	% Female	0.5	-	0.51	-	0.55	0.56	0.61
	Mean Age	39.2	-	42.58	-	50.45	53.86	54.3
Canada	% Female	0.5	0.5	-	0.6	0.58	-	0.49
	Mean Age	39.67	43.05	-	46.33	48.21	-	46.56
Finland	% Female	NA	-	0.51	-	0.52	-	-
	Mean Age	NA	-	42.17	-	47.52	-	-
Hungary	% Female	0.53	-	0.54	-	0.53	-	-
	Mean Age	NA	-	45.15	-	45.55	-	-
Japan	% Female	0.51	0.52	0.51	0.54	0.56	0.52	0.56
	Mean Age	39.97	42.81	44.82	46.85	48.15	50.74	54.78
South Korea	% Female	0.53	0.53	0.51	0.5	0.5	0.51	0.51
	Mean Age	NA	37.39	38.15	39.24	41.38	43.17	45.63
Mexico	% Female	NA	0.45	0.5	0.51	0.51	0.5	0.5
	Mean Age	30.63	34.07	34.73	37.72	39.69	37.48	43.36
South Africa	% Female	NA	0.54	0.5	0.5	0.5	0.5	-
	Mean Age	37.21	37.81	38.53	37.97	38.82	36.67	-
Sweden	% Female	0.48	0.48	0.49	0.5	0.52	0.53	-
	Mean Age	45.14	42.64	44.91	45.19	48.39	47.35	-
United States	% Female	0.55	0.5	0.5	0.58	0.5	0.51	0.46
	Mean Age	40.3	47.33	48.37	42.36	47.96	48.91	43.42
Brazil	% Female	-	0.5	0.5	-	0.58	0.62	0.55
	Mean Age	-	36.38	36.15	-	39.96	42.82	43.56
Belarus	% Female	-	0.54	0.58	-	-	0.55	-
	Mean Age	-	39.75	44.2	-	-	44.42	-
Chile	% Female	-	0.52	0.53	0.53	0.55	0.51	0.53
	Mean Age	-	38.79	39.68	41.45	42.93	43.89	45.27
China	% Female	-	0.4	0.47	0.51	0.54	0.51	0.55
	Mean Age	-	39.28	38.73	40.28	44.72	43.92	44.58
Czechia	% Female	-	0.52	0.57	-	-	-	0.54
	Mean Age	-	43.03	47.9	-	-	-	49.11
India	% Female	-	0.47	0.45	0.43	0.43	0.44	-
	Mean Age	-	35.92	35.92	40.22	41.37	41.24	-
Nigeria	% Female	-	0.4	0.5	0.49	-	0.5	0.49
	Mean Age	-	31.51	32.91	30.68	-	31.22	32.56
Poland	% Female	-	0.51	0.54	-	0.49	0.54	-
	Mean Age	-	43.76	47.16	-	45.96	48.05	-
Russia	% Female	-	0.57	0.59	-	0.53	0.55	0.59
	Mean Age	-	42.76	46.53	-	41.25	46.06	45.41
Slovakia	% Female	-	0.52	0.5	-	-	-	0.54
	Mean Age	-	41.72	44.5	-	-	-	52.66
Spain	% Female	-	0.55	0.52	0.51	0.5	0.51	-

	Mean Age	-	44.13	45.14	46.13	46.21	46.54	-
Switzerland	% Female	-	0.54	0.5	-	0.55	-	-
	Mean Age	-	46.9	45.04	-	52.45	-	-
Turkey	% Female	-	0.5	0.5	0.5	0.5	0.51	0.5
	Mean Age	-	36.41	36.24	36.99	36.48	38.45	38.83
Albania	% Female	-	-	0.51	0.51	-	-	-
	Mean Age	-	-	40.47	40.97	-	-	-
Azerbaijan	% Female	-	-	0.51	-	-	0.5	-
	Mean Age	-	-	36.45	-	-	41.13	-
Bangladesh	% Female	-	-	0.44	0.45	-	-	0.51
	Mean Age	-	-	35.45	33.74	-	-	36.59
Armenia	% Female	-	-	0.53	-	-	0.66	0.69
	Mean Age	-	-	38	-	-	46.59	49.04
Bosnia Herzegovina	% Female	-	-	0.42	0.52	-	-	-
	Mean Age	-	-	41.27	40.65	-	-	-
Bulgaria	% Female	-	-	0.53	-	0.54	-	-
	Mean Age	-	-	46.25	-	47.36	-	-
Taiwan ROC	% Female	-	-	0.51	-	0.49	0.52	0.51
	Mean Age	-	-	41.89	-	43.88	45.48	48.29
Colombia	% Female	-	-	0.49	-	0.5	0.5	0.5
	Mean Age	-	-	36.08	-	36.96	40.41	38.85
Estonia	% Female	-	-	0.56	-	-	0.55	-
	Mean Age	-	-	43.59	-	-	48.57	-
Georgia	% Female	-	-	0.55	-	0.53	0.54	-
	Mean Age	-	-	40.66	-	45.41	44.66	-
Germany	% Female	-	-	0.54	-	0.56	0.5	0.51
	Mean Age	-	-	43.61	-	50.44	49.48	50.8
Moldova	% Female	-	-	0.51	0.56	0.53	-	-
	Mean Age	-	-	43.19	42.77	42.78	-	-
Montenegro	% Female	-	-	0.52	0.5	-	-	-
	Mean Age	-	-	41.9	44.27	-	-	-
New Zealand	% Female	-	-	0.55	-	0.55	0.58	0.57
	Mean Age	-	-	47.44	-	49.25	51.44	57.85
Norway	% Female	-	-	0.51	-	0.5	-	-
	Mean Age	-	-	43.26	-	45.78	-	-
Pakistan	% Female	-	-	0.49	0.48	-	0.48	0.48
	Mean Age	-	-	36.81	36.62	-	34.34	35.65
Peru	% Female	-	-	0.51	0.51	0.51	0.5	0.5
	Mean Age	-	-	34.86	35.5	37.62	39.42	40.16
Philippines	% Female	-	-	0.5	0.5	-	0.5	0.5
	Mean Age	-	-	36.21	38.8	-	42.71	43.71
Puerto Rico	% Female	-	-	0.65	0.64	-	-	0.61
	Mean Age	-	-	42.96	46.32	-	-	49.78
Romania	% Female	-	-	0.51	-	0.54	0.57	0.6
	Mean Age	-	-	42.81	-	48.68	48.39	48.05
Serbia	% Female	-	-	0.51	0.52	0.49	-	0.52
	Mean Age	-	-	44.92	46.34	42.36	-	46.14
Slovenia	% Female	-	-	0.54	-	0.54	0.58	-

	Mean Age	-	-	43.69	-	46.19	49.5	-
Ukraine	% Female	-	-	0.6	-	0.66	0.6	0.59
	Mean Age	-	-	45.8	-	42.38	47.23	47.57
North Macedonia	% Female	-	-	0.48	0.48	-	-	-
	Mean Age	-	-	40.05	42.4	-	-	-
United Kingdom	% Female	-	-	0.54	-	0.51	-	0.57
	Mean Age	-	-	45.79	-	45.69	-	51.82
Uruguay	% Female	-	-	0.59	-	0.56	0.53	0.68
	Mean Age	-	-	46.35	-	46.53	44.99	49.83
Venezuela	% Female	-	-	0.5	0.5	-	-	0.52
	Mean Age	-	-	36.8	36.21	-	-	38.31
Algeria	% Female	-	-	-	0.49	-	0.49	-
	Mean Age	-	-	-	35.58	-	37.8	-
Indonesia	% Female	-	-	-	0.5	0.48	-	0.55
	Mean Age	-	-	-	44.61	36.1	-	40.03
Iran	% Female	-	-	-	0.46	0.5	-	0.49
	Mean Age	-	-	-	34.18	32.69	-	39.48
Iraq	% Female	-	-	-	0.52	0.52	0.48	0.49
	Mean Age	-	-	-	37.2	37.09	36.61	36.6
Jordan	% Female	-	-	-	0.51	0.51	0.5	0.5
	Mean Age	-	-	-	36.06	37.59	39.78	43.31
Kyrgyzstan	% Female	-	-	-	0.55	-	0.51	0.62
	Mean Age	-	-	-	37.04	-	38.75	41.3
Morocco	% Female	-	-	-	0.51	0.51	0.5	0.5
	Mean Age	-	-	-	33.23	37.17	37.26	37.22
Singapore	% Female	-	-	-	0.52	-	0.55	0.54
	Mean Age	-	-	-	33.58	-	41.88	47.78
Vietnam	% Female	-	-	-	0.51	0.49	-	0.55
	Mean Age	-	-	-	42.6	40.75	-	37.89
Zimbabwe	% Female	-	-	-	0.5	-	0.54	0.51
	Mean Age	-	-	-	34.91	-	33.77	39.15
Egypt	% Female	-	-	-	0.49	0.62	0.68	0.48
	Mean Age	-	-	-	37.89	41.02	40.62	39.7
Andorra	% Female	-	-	-	-	0.5	-	0.49
	Mean Age	-	-	-	-	40.65	-	46.83
Cyprus	% Female	-	-	-	-	0.51	0.53	0.52
	Mean Age	-	-	-	-	41.63	42.16	43.54
Ethiopia	% Female	-	-	-	-	0.49	-	0.49
	Mean Age	-	-	-	-	29.93	-	31.93
Ghana	% Female	-	-	-	-	0.49	0.5	-
	Mean Age	-	-	-	-	33.86	30.92	-
Guatemala	% Female	-	-	-	-	0.51	-	0.53
	Mean Age	-	-	-	-	33.75	-	33.5
Hong Kong SAR	% Female	-	-	-	-	0.52	0.55	0.54
	Mean Age	-	-	-	-	44.31	44.67	47.22
Malaysia	% Female	-	-	-	-	0.5	0.49	0.5
	Mean Age	-	-	-	-	31.84	40.01	38.33
Netherlands	% Female	-	-	-	-	0.51	0.54	0.54
	Mean Age	-	-	-	-	44.56	53.34	53.36

Rwanda	% Female	-	-	-	-	0.51	0.5	-
	Mean Age	-	-	-	-	34.65	33.77	-
Thailand	% Female	-	-	-	-	0.51	0.48	0.53
	Mean Age	-	-	-	-	45.35	45.16	46.22
Trinidad Tobago	% Female	-	-	-	-	0.55	0.55	-
	Mean Age	-	-	-	-	42.61	45.87	-
Ecuador	% Female	-	-	-	-	-	0.52	0.52
	Mean Age	-	-	-	-	-	39.81	39.49
Kazakhstan	% Female	-	-	-	-	-	0.6	0.55
	Mean Age	-	-	-	-	-	40.02	41.25
Lebanon	% Female	-	-	-	-	-	0.51	0.5
	Mean Age	-	-	-	-	-	38.37	40.83
Libya	% Female	-	-	-	-	-	0.49	0.48
	Mean Age	-	-	-	-	-	38.42	40.22
Tunisia	% Female	-	-	-	-	-	0.47	0.54
	Mean Age	-	-	-	-	-	38.82	43.16

Note. Dashes indicate that a country was not included in the WVS for a given wave. An NA value indicates that a country was included but the demographic item was not asked (this only occurred for the first wave of the survey).

Supplementary Table 2.
WVS Items Included in Analyses

Item ID	Item Label	Scale
A029	Important child qualities: independence	1 = Mentioned; 0 = Not Mentioned
A030	Important child qualities: Hard work	1 = Mentioned; 0 = Not Mentioned
A032	Important child qualities: feeling of responsibility	1 = Mentioned; 0 = Not Mentioned
A034	Important child qualities: imagination	1 = Mentioned; 0 = Not Mentioned
A035	Important child qualities: tolerance and respect for other people	1 = Mentioned; 0 = Not Mentioned
A038	Important child qualities: thrift saving money and things	1 = Mentioned; 0 = Not Mentioned
A039	Important child qualities: determination perseverance	1 = Mentioned; 0 = Not Mentioned
A040	Important child qualities: religious faith	1 = Mentioned; 0 = Not Mentioned
A041	Important child qualities: unselfishness	1 = Mentioned; 0 = Not Mentioned
A042	Important child qualities: obedience	1 = Mentioned; 0 = Not Mentioned
E069_01	Confidence: Churches	1 (A Great Deal) - 4 (None at All)
E069_02	Confidence: Armed Forces	1 (A Great Deal) - 4 (None at All)
E069_04	Confidence: The Press	1 (A Great Deal) - 4 (None at All)
E069_05	Confidence: Labour Unions	1 (A Great Deal) - 4 (None at All)
E069_06	Confidence: The Police	1 (A Great Deal) - 4 (None at All)
E069_07	Confidence: Parliament	1 (A Great Deal) - 4 (None at All)
E069_08	Confidence: The Civil Services	1 (A Great Deal) - 4 (None at All)
E069_13	Confidence: Major Companies	1 (A Great Deal) - 4 (None at All)
E015	Future changes: Less importance placed on work	1 = Good; 2 = Don't Mind; 3 = Bad
E016	Future changes: More emphasis on technology	1 = Good; 2 = Don't Mind; 3 = Bad
E018	Future changes: Greater respect for authority	1 = Good; 2 = Don't Mind; 3 = Bad
F114A	Justifiable: Claiming government benefits to which you are not entitled	1 (Never Justifiable) - 4 (Always Justifiable)
F115	Justifiable: Avoiding a fare on public transport	1 (Never Justifiable) - 4 (Always Justifiable)
F116	Justifiable: Cheating on taxes	1 (Never Justifiable) - 10 (Always Justifiable)
F117	Justifiable: Someone accepting a bribe	1 (Never Justifiable) - 10 (Always Justifiable)
F118	Justifiable: Homosexuality	1 (Never Justifiable) - 10 (Always Justifiable)
F119	Justifiable: Prostitution	1 (Never Justifiable) - 10 (Always Justifiable)
F120	Justifiable: Abortion	1 (Never Justifiable) - 10 (Always Justifiable)
F121	Justifiable: Divorce	1 (Never Justifiable) - 10 (Always Justifiable)
F122	Justifiable: Euthanasia	1 (Never Justifiable) - 10 (Always Justifiable)

F123	Justifiable: Suicide	1 (Never Justifiable) - 10 (Always Justifiable)
A165	Most people can be trusted	1 = Can be Trusted; 2 = Need to be Very Careful
E033	Self positioning in political scale	1 (Left) - 10 (Right)
G006	How proud of nationality	1 (Very Proud) - 10 (Not at all Proud)
A124_02	Neighbours: People of a different race	1 = Mentioned; 0 = Not Mentioned
A124_03	Neighbours: Heavy drinkers	1 = Mentioned; 0 = Not Mentioned
A124_06	Neighbours: Immigrants/foreign workers	1 = Mentioned; 0 = Not Mentioned
E025	Political action: Signing a petition	1 = Have Done; 1 = Might Do; 2 = Would Never Do
E026	Political action: joining in boycotts	1 = Have Done; 1 = Might Do; 2 = Would Never Do
E027	Political action: attending lawful/peaceful demonstrations	1 = Have Done; 1 = Might Do; 2 = Would Never Do

Supplementary Table 3.
WVS Items According to Change in Means

Item Label	Original Scale	Trend
Confidence: Armed Forces	1 (A Great Deal) - 4 (None at All)	-0.86
Future changes: Less importance placed on work	1 = Good; 2 = Don't Mind; 3 = Bad	-0.77
Future changes: Greater respect for authority	1 = Good; 2 = Don't Mind; 3 = Bad	-0.75
How proud of nationality	1 (Very Proud) - 10 (Not at all Proud)	-0.74
Justifiable: Abortion	1 (Never Justifiable) - 10 (Always Justifiable)	-0.56
Justifiable: Euthanasia	1 (Never Justifiable) - 10 (Always Justifiable)	-0.41
Confidence: Churches	1 (A Great Deal) - 4 (None at All)	-0.40
Justifiable: Cheating on taxes	1 (Never Justifiable) - 10 (Always Justifiable)	-0.39
Future changes: More emphasis on technology	1 = Good; 2 = Don't Mind; 3 = Bad	-0.26
Self positioning in political scale	1 (Left) - 10 (Right)	-0.16
Justifiable: Suicide	1 (Never Justifiable) - 10 (Always Justifiable)	0.02
Confidence: The Police	1 (A Great Deal) - 4 (None at All)	0.06
Justifiable: Divorce	1 (Never Justifiable) - 10 (Always Justifiable)	0.19
Important child qualities: thrift saving money and things	1 = Mentioned; 0 = Not Mentioned	0.36
Important child qualities: tolerance and respect for other people	1 = Mentioned; 0 = Not Mentioned	0.47
Important child qualities: obedience	1 = Mentioned; 0 = Not Mentioned	0.48
Important child qualities: feeling of responsibility	1 = Mentioned; 0 = Not Mentioned	0.49
Confidence: Major Companies	1 (A Great Deal) - 4 (None at All)	0.49
Important child qualities: determination perseverance	1 = Mentioned; 0 = Not Mentioned	0.53
Political action: attending lawful/peaceful demonstrations	1 = Have Done; 1 = Might Do; 2 = Would Never Do	0.53
Neighbours: People of a different race	1 = Mentioned; 0 = Not Mentioned	0.57
Important child qualities: imagination	1 = Mentioned; 0 = Not Mentioned	0.60
Important child qualities: religious faith	1 = Mentioned; 0 = Not Mentioned	0.60
Important child qualities: independence	1 = Mentioned; 0 = Not Mentioned	0.61
Justifiable: Someone accepting a bribe	1 (Never Justifiable) - 10 (Always Justifiable)	0.61
Justifiable: Prostitution	1 (Never Justifiable) - 10 (Always Justifiable)	0.61
Important child qualities: Hard work	1 = Mentioned; 0 = Not Mentioned	0.63
Confidence: Labour Unions	1 (A Great Deal) - 4 (None at All)	0.74
Important child qualities: unselfishness	1 = Mentioned; 0 = Not Mentioned	0.75
Justifiable: Avoiding a fare on public transport	1 (Never Justifiable) - 10 (Always Justifiable)	0.77

Neighbours: Immigrants/foreign workers	1 = Mentioned; 0 = Not Mentioned	0.79
Justifiable: Homosexuality	1 (Never Justifiable) - 10 (Always Justifiable)	0.79
Neighbours: Heavy drinkers	1 = Mentioned; 0 = Not Mentioned	0.82
Confidence: The Press	1 (A Great Deal) - 4 (None at All)	0.82
Confidence: The Civil Services	1 (A Great Deal) - 4 (None at All)	0.84
Justifiable: Claiming government benefits to which you are not entitled	1 (Never Justifiable) - 10 (Always Justifiable)	0.85
Political action: joining in boycotts	1 = Have Done; 1 = Might Do; 2 = Would Never Do	0.89
Confidence: Parliament	1 (A Great Deal) - 4 (None at All)	0.90
Most people can be trusted	1 = Can be Trusted; 2 = Need to be Very Careful	0.91
Political action: Signing a petition	1 = Have Done; 1 = Might Do; 2 = Would Never Do	0.93

Note. Trends are derived from Pearson correlations assessing the relationship between timepoint (1-7) and the normalized mean. The original scale was converted to a 0 – 1 index, where values approaching 1 indicated higher-value responses on the original scale.

**Supplementary Table 4.**<br>WVS Items According to Value Variation Trends				
**Item Label**	**Correlation by Coverage Subset**			
	2 Waves	3 Waves	4 Waves	5 Waves
Justifiable: Homosexuality	0.96	0.96	0.94	0.96
Justifiable: Euthanasia	0.96	0.84	0.87	0.76
Justifiable: Divorce	0.92	0.89	0.85	0.87
Justifiable: Prostitution	0.91	0.85	0.82	0.85
Justifiable: Suicide	0.89	0.78	0.77	0.81
Important child qualities: obedience	0.87	0.80	0.69	0.52
Justifiable: Avoiding a fare on public transport	0.87	0.79	0.93	0.88
Justifiable: Abortion	0.87	0.81	0.84	0.87
Important child qualities: religious faith	0.79	0.76	0.77	0.35
Neighbours: Immigrants/foreign workers	0.75	0.76	0.62	0.55
Important child qualities: determination perseverance	0.73	0.74	0.82	0.83
Justifiable: Someone accepting a bribe	0.72	0.73	0.79	0.29
Most people can be trusted	0.70	0.68	0.78	0.81
Political action: attending lawful/peaceful demonstrations	0.69	0.73	0.63	0.33
Political action: Signing a petition	0.67	0.61	0.77	0.64
Neighbours: People of a different race	0.63	0.57	0.32	0.33
Confidence: Labour Unions	0.48	0.56	0.44	0.48
Confidence: Parliament	0.43	0.39	0.56	0.74
Important child qualities: imagination	0.42	0.49	0.57	0.70
How proud of nationality	0.39	0.28	0.10	-0.22
Confidence: The Press	0.34	0.41	0.78	0.79
Political action: joining in boycotts	0.29	0.31	0.33	0.26
Important child qualities: unselfishness	0.23	0.20	0.22	0.19
Confidence: Churches	0.19	0.10	0.05	-0.05
Important child qualities: independence	0.14	0.23	0.29	0.19
Confidence: The Civil Services	0.13	0.12	-0.07	0.32
Confidence: The Police	0.07	0.36	0.60	0.49
Important child qualities: Hard work	-0.02	0.04	-0.06	0.00
Justifiable: Claiming government benefits to which you are not entitled	-0.03	0.76	0.64	0.66
Important child qualities: tolerance and respect for other people	-0.04	-0.08	-0.17	-0.33
Self positioning in political scale	-0.06	0.01	0.01	-0.74
Neighbours: Heavy drinkers	-0.24	-0.17	0.27	0.52
Important child qualities: thrift saving money and things	-0.26	-0.25	-0.17	-0.04
Confidence: Major Companies	-0.32	-0.34	-0.61	-0.74
Justifiable: Cheating on taxes	-0.40	-0.26	0.16	0.01
Confidence: Armed Forces	-0.45	-0.50	-0.69	-0.72
Future changes: Less importance placed on work	-0.57	-0.59	-0.17	-0.24

Future changes: Greater respect for authority	-0.73	-0.72	-0.68	-0.62
Important child qualities: feeling of responsibility	-0.74	-0.84	-0.71	-0.58
Future changes: More emphasis on technology	-0.91	-0.91	-0.94	-0.91
**Median Correlation**	**0.37**	**0.40**	**0.50**	**0.34**

Note. The sets of columns refer to samples of countries that have participated in *n* waves. For example, the figures reported in the “5 Waves” column have been computed on a sub-sample of countries that have participated in at least 5 of the 7 WVS waves. We report figures across these sub-samples for the sake of transparency and robustness.

Supplementary Table 5.
Value Distinctiveness by Country and WVS Wave

Country	Waves	Value Distinctiveness by Wave						
		1	2	3	4	5	6	7
Albania	2	-	-	0.10	0.07	-	-	-
Algeria	2	-	-	-	0.10	-	0.07	-
Andorra	2	-	-	-	-	0.15	-	0.13
Argentina	7	0.09	0.09	0.09	0.10	0.10	0.11	0.09
Armenia	3	-	-	0.07	-	-	0.10	0.10
Australia	5	0.07	-	0.10	-	0.12	0.15	0.14
Azerbaijan	2	-	-	0.08	-	-	0.11	-
Bangladesh	3	-	-	0.16	0.18	-	-	0.13
Belarus	3	-	0.09	0.07	-	-	0.08	-
Bosnia Herzegovina	2	-	-	0.06	0.07	-	-	-
Brazil	5	-	0.07	0.10	-	0.09	0.08	0.09
Bulgaria	2	-	-	0.07	-	0.09	-	-
Canada	5	0.04	0.08	-	0.11	0.09	-	0.14
Chile	6	-	0.10	0.07	0.07	0.07	0.08	0.09
China	6	-	0.13	0.11	0.13	0.14	0.12	0.14
Colombia	4	-	-	0.09	-	0.10	0.11	0.09
Cyprus	3	-	-	-	-	0.06	0.06	0.06
Ecuador	2	-	-	-	-	-	0.09	0.08
Egypt	4	-	-	-	0.15	0.14	0.11	0.14
Estonia	2	-	-	0.08	-	-	0.09	-
Ethiopia	2	-	-	-	-	0.10	-	0.11
Finland	3	0.07	-	0.11	-	0.12	-	-
Georgia	3	-	-	0.08	-	0.10	0.10	-
Germany	4	-	-	0.16	-	0.12	0.14	0.16
Ghana	2	-	-	-	-	0.13	0.13	-
Guatemala	2	-	-	-	-	0.12	-	0.10
Hong Kong SAR	3	-	-	-	-	0.17	0.10	0.09
Hungary	3	0.11	-	0.08	-	0.09	-	-
India	5	-	0.10	0.11	0.08	0.10	0.10	-
Indonesia	3	-	-	-	0.12	0.13	-	0.12
Iran	3	-	-	-	0.11	0.10	-	0.11
Iraq	4	-	-	-	0.14	0.16	0.09	0.10
Japan	7	0.09	0.11	0.13	0.18	0.16	0.15	0.16
Jordan	4	-	-	-	0.13	0.19	0.11	0.12
Kazakhstan	2	-	-	-	-	-	0.08	0.10
Kyrgyzstan	3	-	-	-	0.08	-	0.09	0.09
Lebanon	2	-	-	-	-	-	0.09	0.10
Libya	2	-	-	-	-	-	0.11	0.12
Malaysia	3	-	-	-	-	0.12	0.12	0.09
Mexico	7	0.10	0.08	0.07	0.08	0.08	0.09	0.08

Moldova	3	-	-	0.08	0.09	0.09	-	-
Montenegro	2	-	-	0.05	0.10	-	-	-
Morocco	4	-	-	-	0.12	0.09	0.09	0.07
Netherlands	2	-	-	-	-	0.12	0.15	0.17
New Zealand	4	-	-	0.12	-	0.12	0.14	0.16
Nigeria	5	-	0.14	0.13	0.12	-	0.09	0.12
North Macedonia	2	-	-	0.10	0.09	-	-	-
Norway	2	-	-	0.15	-	0.17	-	-
Pakistan	4	-	-	0.13	0.14	-	0.10	0.13
Peru	5	-	-	0.10	0.08	0.13	0.09	0.07
Philippines	4	-	-	0.12	0.10	-	0.11	0.12
Poland	4	-	0.09	0.07	-	0.06	0.07	-
Puerto Rico	3	-	-	0.10	0.06	-	-	0.09
Romania	4	-	-	0.07	-	0.10	0.07	0.08
Russia	5	-	0.07	0.08	-	0.09	0.09	0.08
Rwanda	2	-	-	-	-	0.15	0.09	-
Serbia	4	-	-	0.06	0.09	0.11	-	0.09
Singapore	3	-	-	-	0.07	-	0.10	0.08
Slovakia	2	-	0.13	0.07	-	-	-	0.09
Slovenia	3	-	-	0.08	-	0.10	0.13	-
South Africa	6	0.06	0.10	0.09	0.07	0.08	0.10	-
South Korea	7	0.10	0.11	0.11	0.14	0.11	0.10	0.11
Spain	5	-	0.08	0.09	0.10	0.09	0.10	-
Sweden	6	0.10	0.13	0.18	0.22	0.18	0.19	-
Switzerland	3	-	0.11	0.12	-	0.17	-	-
Taiwan ROC	4	-	-	0.08	-	0.11	0.10	0.10
Thailand	3	-	-	-	-	0.11	0.09	0.09
Trinidad and Tobago	2	-	-	-	-	0.10	0.11	-
Tunisia	2	-	-	-	-	-	0.09	0.09
Turkey	6	-	0.09	0.13	0.09	0.09	0.07	0.08
Ukraine	4	-	-	0.06	-	0.09	0.07	0.08
United States	7	0.05	0.07	0.08	0.10	0.08	0.09	0.10
Uruguay	3	-	-	0.08	-	0.10	0.11	0.12
Venezuela	3	-	-	0.10	0.09	-	-	0.08
Vietnam	3	-	-	-	0.14	0.19	-	0.16
Zimbabwe	3	-	-	-	0.11	-	0.09	0.10

Note. Dashes indicate waves where a country was not included in the WVS.

Supplementary Table 6.
Value Distinctiveness by Country and Decade

**Country**	**Value Distinctiveness by Decade**		
Country	1990 – 1999	2000 – 2009	2010 – 2020
Argentina	0.09	0.10	0.08
Australia	0.10	0.13	0.13
Bangladesh	0.16	0.21	0.16
Brazil	0.08	0.08	0.08
Canada	0.09	0.09	0.13
Chile	0.08	0.06	0.06
China	0.13	0.14	0.14
Taiwan ROC	0.09	0.11	0.09
Colombia	0.08	0.09	0.10
Georgia	0.07	0.10	0.13
Germany	0.17	0.12	0.12
India	0.10	0.09	0.11
Japan	0.12	0.16	0.14
South Korea	0.12	0.12	0.09
Mexico	0.07	0.07	0.08
New Zealand	0.12	0.12	0.12
Nigeria	0.12	0.15	0.12
Pakistan	0.13	0.16	0.13
Peru	0.09	0.10	0.09
Philippines	0.12	0.11	0.13
Poland	0.07	0.06	0.06
Puerto Rico	0.09	0.08	0.08
Romania	0.08	0.10	0.10
Russia	0.08	0.09	0.09
Serbia	0.08	0.08	0.09
Slovenia	0.08	0.11	0.10
South Africa	0.07	0.07	0.10
Spain	0.08	0.08	0.08
Sweden	0.17	0.19	0.17
Turkey	0.09	0.09	0.10
Ukraine	0.07	0.09	0.08
United States	0.07	0.07	0.08
Uruguay	0.08	0.10	0.09

Supplementary Table 7.
Second-Order Polynomial Model of CF_{ST} Over Time

	b (SE)	*t* value	*df*	*p* value	95% *CI*s
Timepoint (Linear)	0.02 (.003)	7.42	6,041	< 0.001	.02, .03
Timepoint (Quadratic)	-0.002 (.0003)	-6.61	6,042	< 0.001	-0.003, -0.002

Supplementary Table 8.
Associations with Value Distinctiveness in Multiple Regression Without GDP Per Capita

	b (SE)	*t* value	*df*	*p* value	95% *CI*s
Timepoint $_{(C)}$	-0.002 (0.004)	-0.65	71.12	0.518	-0.01, 0.01
Timepoint $_{(L)}$	0.002 (0.001)	1.88	869.00	0.060	-0.0001, 0.004
Political Rights	-0.01 (0.01)	-1.52	2028.34	0.129	-0.02, 0.003
Gini	0.03 (0.02)	1.56	142.18	0.121	-0.01, 0.06
Distance from Equator	-0.005 (0.01)	-0.41	51.35	0.685	-0.03, 0.02
Globalization	0.02 (0.02)	1.08	407.49	0.280	-0.02, 0.05

Supplementary Table 9.
Associations with Value Distinctiveness in Multiple Regression

	b (SE)	*t* value	*df*	*p* value	95% *CI*s
Timepoint $_{(C)}$	0.0004 (0.004)	0.09	63.57	0.925	-0.01, 0.01
Timepoint $_{(L)}$	-0.003 (0.002)	-2.07	10045.68	0.038	-0.01, -0.0002
GDP Per Capita $_{(L)}$	0.03 (0.01)	6.82	10045.71	< 0.001	0.02, 0.04
GDP Per Capita $_{(C)}$	0.05 (0.02)	2.73	64.32	0.008	0.01, 0.08
Gini $_{(L)}$	0.003 (0.003)	1.26	10045.61	0.209	-0.002, 0.01
Gini $_{(C)}$	-0.003 (0.03)	-0.11	31.51	0.916	-0.05, 0.05
Political Rights $_{(L)}$	-0.01 (0.003)	-1.77	10045.63	0.077	-0.01, 0.001
Political Rights $_{(C)}$	0.0003 (0.02)	0.02	65.59	0.983	-0.04, 0.03
Globalization $_{(L)}$	0.01 (0.01)	1.45	10045.66	0.146	-0.003, 0.02
Globalization $_{(C)}$	-0.02 (0.04)	-0.57	62.51	0.571	-0.09, 0.05
Distance from Equator	-0.01 (0.01)	-0.85	63.67	0.400	-0.03, 0.01

Note. Subscripts "(C)" and "(L)" stand for variables which represent country means or have been centered within country means to indicate how the same countries are changing over time. Equator is not separated because it does not vary over time within countries.

Supplementary Table 10.
Fit Statistics Associated with Non-Linear Models

Model	AIC	BIC
Value Variation (Linear)	-1134.74	-1120.20
**Value Variation (Quadratic)**	**-1155.70**	**-1137.53**
Value Variation (Spline_a)	-1149.93	-1131.51
Value Variation (Spline_b)	-1150.02	-1131.51
Value Variation (Spline_c)	-1149.69	-1131.51
Value Distinctiveness (Linear)	-22073.62	-22029.99
**Value Distinctiveness (Quadratic)**	**-22111.82**	**-22060.92**
Value Distinctiveness (Spline_a)	-22100.88	-22049.98
Value Distinctiveness (Spline_b)	-22100.25	-22049.34
Value Distinctiveness (Spline_c)	-22098.75	-22047.85

Note. The best-fitting model for each outcome variable is bolded here.

Supplementary Table 11.
Results of Non-Linear Models

	b (SE)	*t* value	*df*	*p* value	95% *CI*s
**Value Variation**					
Linear Slope	0.13 (0.02)	5.35	238	< 0.001	0.08, 0.17
Quadratic Slope	-0.11 (0.02)	-4.74	238	< 0.001	-0.16, -0.07
**Value Distinctiveness**					
Linear Slope	0.48 (0.10)	5.07	9,391	< 0.001	0.30, 0.67
Quadratic Slope	-0.51 (0.09)	-5.74	10,560	< 0.001	-0.68, -0.33

Supplementary Table 12.
Associations with Value Distinctiveness in Multiple Regression

	b (SE)	*t* value	*df*	*p* value	95% *CI*s
Timepoint $_{(C)}$	-0.003 (0.003)	-0.77	77.18	0.445	-0.01, 0.005
Timepoint $_{(L)}$	-0.001 (0.001)	-0.79	412.55	0.431	-0.003, 0.001
Within-Country Hetero.	-0.06 (0.01)	-4.45	8031.68	< 0.001	-0.09, -0.03
GDP Per Capita	0.08 (0.01)	7.96	590.55	< 0.001	0.06, 0.09
Gini	0.03 (0.02)	1.71	204.96	0.088	-0.004, 0.06
Globalization	0.003 (0.02)	0.21	532.26	0.835	-0.03, 0.03
Political Rights	-0.01 (0.01)	-1.84	1972.73	0.066	-0.02, 0.001
Distance from Equator	-0.01 (0.01)	-0.92	64.43	0.363	-0.03, 0.01

Supplementary Table 13.
Associations with Value Distinctiveness in Multiple Regression

	b (SE)	*t* value	*df*	*p* value	95% *CI*s
Timepoint $_{(C)}$	0.001 (0.01)	0.45	52.65	0.656	-0.005, 0.01
Timepoint $_{(L)}$	0.00003 (0.003)	0.04	628.69	0.968	-0.002, 0.001
GDP Per Capita in Europe	0.08 (0.02)	3.63	18.76	0.002	0.04, 0.12
GDP Per Capita in Asia	-0.02 (0.03)	-0.77	16.24	0.448	-0.08, 0.03
GDP Per Capita in Africa	-0.14 (0.10)	-1.38	109.20	0.171	-0.32, 0.06
Continent: Oceania	-0.01 (0.02)	-0.39	94.78	0.701	-0.04, 0.03
Continent: N America	-0.003 (0.01)	-0.29	72.84	0.775	-0.02, 0.02
Continent: S America	0.01 (0.01)	0.94	66.40	0.352	-0.01, 0.03
Continent: Africa	0.03 (0.01)	3.35	59.62	0.001	0.01, 0.05
Continent: Asia	0.03 (0.01)	4.33	58.91	< 0.001	0.02, 0.05
GDP × Europe: Oceania	0.03 (0.06)	0.61	11.21	0.551	-0.07, 0.14
GDP × Europe: N America	-0.03 (0.04)	-0.65	14.94	0.527	-0.12, 0.05
GDP × Europe: S America	-0.06 (0.05)	-1.15	52.75	0.254	-0.16, 0.04
GDP × Europe: Africa	-0.21 (0.10)	-2.13	122.58	0.035	-0.40, -0.02
GDP × Europe: Asia	-0.10 (0.03)	-2.84	16.66	0.011	-0.16, -0.04

Note. These coefficients come from a mixed effects model where GDP per capita is interacted with continent, predicting value distinctiveness.

Supplementary Table 14.
Associations Between Value Dimensions

	Secular	Emancipative	Post-Materialist
Secular	-		
Emancipative	0.56 (< 0.001)	-	
Post-Materialist	0.14 (0.022)	0.63 (< 0.001)	-

Note. P-values are provided in parentheses.

Supplementary Table 15.
Associations Between Principal Components and Previously Established Value Dimensions

	b (SE)	*t* value	*df*	*p* value	95% *CI*s
**PC1**					
Secular	0.26 (0.03)	8.60	233.90	< 0.001	0.20, 0.32
Emancipative	0.76 (0.04)	19.46	215.60	< 0.001	0.68, 0.85
Post-Materialist	-0.06 (0.03)	-2.12	246.80	0.035	-0.12, -0.005
**PC2**					
Secular	-0.13 (0.07)	-2.05	194.81	0.042	-0.27, 0.01
Emancipative	0.11 (0.09)	1.27	125.37	0.208	-0.06, 0.28
Post-Materialist	0.01 (0.06)	0.17	213.50	0.866	-0.11, 0.13

Supplementary Table 16.
Examples of Diverging Values and Notable Countries

Item Label	% Change in Variation	Example of Diverging Countries
Important Child Qualities: Obedience	+41.86%	Dropped in Moldova from 0.40/1.00 to 0.16/1.00<br><br>Rose in Venezuela from 0.48/1.00 to 0.69/1.00
Justifiable: Divorce	+105.85%	Dropped in Kyrgyzstan from 0.27/1.00 to 0.14/1.00<br><br>Rose in Chile from 0.28/1.00 to 0.57/1.00
Important Child Qualities: Religious Faith	+99.49%	Dropped in Japan from 0.05/1.00 to 0.04/1.00<br><br>Rose in Armenia from 0.12/1.00 to 0.38/1.00
Neighbors: Immigrants, /Foreign Workers<br><br>(Item Reflects Aversion to Having as Neighbor)	+349.51%	Dropped in Indonesia from 0.33/1.00 to 0.17/1.00<br><br>Rose in Iran from 0.09/1.00 to 0.42/1.00
Justifiable: Euthanasia	+60.89%	Dropped in Turkey from 0.22/1.00 to 0.17/1.00<br><br>Rose in Spain from 0.39/1.00 to 0.51/1.00

Note. The "% Change in Variation" variable is calculated by taking the absolute difference of the standard deviation of country means between timepoint 1 and timepoint 7, and then dividing it by the standard deviation at timepoint 1. In this equation, 100% change represents an item where variation across countries has doubled over time.

Supplementary Table 17.
Full Coefficients from Clustering Analysis Regressions

	b (SE)	*t* value	*df*	*p* value	95% *CI*s
**Timepoint 1**					
Geography	0.16 (0.13)	1.30	42.49	0.202	-0.09, 0.40
GDP Per Capita	0.46 (0.20)	2.24	46.06	0.030	0.07, 0.84
Gini	0.07 (0.13)	0.55	46.26	0.586	-0.18, 0.32
Religion	-0.001 (0.19)	-0.01	27.46	0.994	-0.40, 0.38
Political Rights	-0.19 (0.20)	-0.94	45.66	0.354	-0.57, 0.20
**Timepoint 2**					
Geography	0.17 (0.08)	2.15	110.20	0.034	0.02, 0.33
GDP Per Capita	0.55 (0.07)	7.70	103.60	< 0.001	0.41, 0.69
Gini	0.10 (0.07)	1.35	106.35	0.179	-0.04, 0.24
Religion	0.35 (0.09)	3.96	96.77	< 0.001	0.17, 0.52
Political Rights	0.20 (0.07)	2.77	111.93	0.007	0.06, 0.34
**Timepoint 3**					
Geography	0.13 (0.03)	4.36	935.98	< 0.001	0.07, 0.19
GDP Per Capita	0.55 (0.03)	16.58	848.19	< 0.001	0.48, 0.61
Gini	0.17 (0.02)	7.36	918.12	< 0.001	0.13, 0.22
Religion	0.11 (0.03)	3.51	920.24	< 0.001	0.05, 0.17
Political Rights	0.10 (0.03)	3.75	939.95	< 0.001	0.05, 0.16
**Timepoint 4**					
Geography	0.23 (0.04)	6.37	544.77	< 0.001	0.16, 0.30
GDP Per Capita	0.41 (0.04)	9.40	538.98	< 0.001	0.32, 0.49
Gini	0.11 (0.03)	3.85	522.29	< 0.001	0.05, 0.16
Religion	0.12 (0.03)	4.18	521.45	< 0.001	0.06, 0.18
Political Rights	0.10 (0.03)	3.43	516.68	< 0.001	0.04, 0.16
**Timepoint 5**					
Geography	0.23 (0.02)	9.50	1116.78	< 0.001	0.19, 0.28
GDP Per Capita	0.56 (0.03)	21.53	1121.06	< 0.001	0.50, 0.61
Gini	0.08 (0.02)	4.18	1085.17	< 0.001	0.04, 0.12
Religion	0.17 (0.02)	7.02	1119.00	< 0.001	0.12, 0.21
Political Rights	0.07 (0.03)	2.59	1115.81	0.010	0.02, 0.12
**Timepoint 6**					
Geography	0.30 (0.03)	12.07	1220.00	< 0.001	0.25, 0.35
GDP Per Capita	0.56 (0.03)	21.31	1184.44	< 0.001	0.51, 0.61
Gini	0.05 (0.02)	2.47	1268.61	0.014	0.01, 0.10
Religion	0.11 (0.02)	5.11	1265.24	< 0.001	0.07, 0.15
Political Rights	0.10 (0.02)	5.01	1239.49	< 0.001	0.06, 0.14
**Timepoint 7**					
Geography	0.35 (0.03)	13.44	1070.11	< 0.001	0.30, 0.40
GDP Per Capita	0.59 (0.03)	19.95	973.60	< 0.001	0.53, 0.64
Gini	0.05 (0.03)	1.82	1039.74	0.069	-0.004, 0.10
Religion	0.15 (0.02)	6.80	1064.27	< 0.001	0.10, 0.19
Political Rights	0.07 (0.02)	3.08	1058.23	0.002	0.03, 0.12

Supplementary Methods

Extended Materials and Methods

The World Values Survey. The World Values Survey (WVS) is an international research program devoted to measuring the social, political, economic, and religious values of individuals around the world. The WVS was founded in 1981 by Inglehart, and has since conducted surveys around the world in "waves" which are designed to operate every five years. The WVS is a unique source of data not only because of its geographic and thematic scope, but also because it is publicly available without any cost. Because of these qualities, the WVS has become the largest non-commercial cross-national empirical time-series investigation of human beliefs and values, with over 60,000 citations on Google Scholar and over 800,000 annual downloads[1].

The WVS collects data from across nations using a global network of social scientists studying changing values and their impact on social and political life. The WVS website contains comprehensive information about its research procedures (https://www.worldvaluessurvey.org). This includes information about translation procedures, and fieldwork training. The survey is delivered through face-to-face interviews at the respondent's place of residence, and responses are either transcribed on pen-and-paper questionnaires or by the CAPI (Computer-Assisted Personal Interview) software, and a minimum number of 1200 completed interviews are required per country. The WVS requires a complete explanation of proposed sampling procedures before the start of fieldwork, and the sampling plan must be approved in writing.

In addition to publishing data each wave, the WVS publishes a time-series file containing data from all waves. The WVS has not surveyed the same people over time in this file. Rather, each timepoint contains a demographically representative snapshot of people in a nation at a particular point in time. The WVS also publishes a list of variables indicating which items are asked in different waves, and a list of nations indicating which nations are surveyed in each wave. The timeseries dataset is published in many different formats. We downloaded the Rdata format.

Characteristics of Countries. We focused on the 76 countries where the WVS has collected data for at least two waves, a necessary condition since we are interested in change over time. Supplementary Table 1 summarizes the age-sex composition of each country in each wave, with dashes indicating waves where a country was not included in data collection.

Samples are designed to be representative of people age 18 and older residing within private residences in each country, regardless of their citizenship or language. The WVS employs probability sampling and stratified sampling to achieve these targets, but they also offer case weights to compensate for small deviations in the resulting sample with respect to gender-age (self-reported), rural-urban, or education. We employed these case weights when they calculated our value means, but our results were substantively identical regardless of whether we use the weights. We report these alternative results later in the supplementary materials.

There are some important changes to the WVS composition over time. The first wave was the smallest, with only 11 countries. It also contains the greatest proportion of wealthy nations. This limitation is the primary reason why we replicate our results with different subsets of nations which have participated in different a larger proportion of waves, and also replicate our results with a stable sample of nations which has participated in three decades of data collection. These analyses are less sensitive to the composition of specific waves.

Characteristics of Items. The WVS contains a heterogeneous set of items that change each year. Researchers can purchase items in a given WVS wave, which means that many items are only included in a single wave. Of the 1011 items included in the WVS timeseries file, 542 items were asked in a single wave, 186 items were asked in two waves, 67 items were asked in three waves, 34 items were asked in four waves, 56 items were asked in five waves, 41 items were asked in six waves, and 85 items were asked in all seven waves.

We focused on the items that were asked in all seven waves, and specifically on items that could be construed broadly as values. This involved removing items were either procedural questions for the WVS administrators (e.g., Year/month of fieldwork), meta-data for analysts (e.g., country code), or demographic items (e.g., Sex, Year of birth). We also removed items which asked participants to report on their own personal qualities, such as their life satisfaction (e.g., health, happiness, satisfaction with employment), life goals (e.g., aims in life), and their personal religiosity (e.g., denomination, service attendance). However, we retained items asking participants about their perceived importance of political action (e.g., signing a petition) and their perceived importance of religion for society (e.g., confidence in the church, religious faith as an important child quality) and whether participants would consider taking part in political activities. In total, we selected 40 items for analysis. In Table S2, we provide the item identification number, item label, and the scale that participants used to respond to the item. Our OSF page contains item information for all 1011 items included in the WVS timeseries. This information is also provided on the WVS website.

We note that these item labels are not the exact text that participants saw. For example, the items with labels beginning with “justifiable” were asked using the following wording:

“*Please tell me for each of the following actions whether you think it can always be justified, never be justified, or something in between, using this card” (read out and code one answer for each statement)*

Readers can access the complete item wording for each item by downloading one of the PDF questionnaires for any wave from the WVS website, since these items were included in each wave.

One of the scales in our analysis involved the qualities that respondents felt to be important for children to learn. For these items, respondents were not allowed to select more than 5 items. However, during data processing we realized that this rule was not always followed. To keep the questionnaire format consistent across countries and waves, we excluded all respondents (n = 20,380; 5% of the total sample) who indicated more than 5 important childhood qualities prior to data analysis. This decision did not affect our results. All results replicated with or without excluding participants who did not follow instructions.

Exogenous Variables. In addition to our WVS data, we also collected data on exogenous variables illustrating the geopolitical conditions of nations over time. We sought to match all exogenous variables as closely as possible to the year of WVS data collection, so we downloaded time-varying estimates of these variables. In some cases, data were not available for a specific country in a specific year. In this case, we used the closest available value—an approach known as Last Observation Carried Forward (LOCF) imputation—and noted the number of years in which this value deviated from the year of WVS data collection in our dataframe.

GDP Per Capita. We accessed GDP per Capita (current USD) from the World Bank. The access URL is https://data.worldbank.org/indicator/NY.GDP.MKTP.CD. The World Bank provides the following description of the variable: “GDP at purchaser's prices is the sum of gross value added by all resident producers in the economy plus any product taxes and minus any subsidies not included in the value of the products. It is calculated without making deductions for depreciation of fabricated assets or for depletion and degradation of natural resources. Data are in current U.S. dollars. Dollar figures for GDP are converted from domestic currencies using single year official exchange rates. For a few countries where the official exchange rate does not reflect the rate effectively applied to actual foreign exchange transactions, an alternative conversion factor is used.”

Gini. We accessed Gini coefficients from the World Inequality Database (WID). The access URL is https://wid.world/data/. The WID has a detailed overview of their data collection methodology, which is available at https://wid.world/methodology/. Each year, they publish a report which summarizes their most recent data collection patterns. The most recent report is available at https://wir2022.wid.world/.

Globalization. We measured globalization using the widely used KOF index published by the Swiss Economic Institute. We accessed time-varying estimates of globalization using the “Global Economy” database, which is a subscription-based service offering time-varying geopolitical data. The access URL is https://www.theglobaleconomy.com/download-data.php. We note that there are actually four globalization indices published by the KOF: An “overall globalization” index, and then indices for political, economic, and social globalization. These indices are in turn made up of individual indicators, such as international debt (economic globalization), migration (social globalization), and number of foreign embassies (political globalization). The full list of indicators, weighting procedure, and more details about methodology are available at the access URL, within PDF reports that are published by the Swiss Economic Institute. The political, social, and economic sub-indices correlate between 0.26 and 0.80. We considered using different sub-indices in our analyses. However, we found that results were similar across the three sub-indices, so we focused on the overall globalization index. We have included each index within the data published on our OSF project page, so that others can explore effects across the indices.

Political Rights. We measured political rights using the “Political Rights” index published by the Freedom House. We accessed this variable using the “Global Economy” database. The access URL is https://www.theglobaleconomy.com/download-data.php. The index is developed by a team of in-house and external analysts and expert advisors who consider a large number of external factors, including participators democracy, media freedom, civil liberties to develop overall scores. The methodology for this index summarized in depth here: https://freedomhouse.org/reports/freedom-world/freedom-world-research-methodology.

We note that the Freedom House actually publishes two different indices: A political rights index and a civil liberties index. Both indices are coded on a 7-point scale from 1 (Strong) to 7 (Weak). These correlated at 0.94 and showed identical results, so we focused on the political rights index in our analyses. We recoded the index so that higher values meant more political rights.

Previous papers have measured democratization using other variables, such as the “Democracy Index” published by the Economist[2]. We used the Freedom House Political Rights index because it was publicly available and time-varying over the course of our study, whereas other indices were not publicly available or were published at specific points in time. The Political Rights index also appears to be face valid and high-quality. According to the Freedom

House website (https://freedomhouse.org/report/freedom-world), "Since 1973, the Freedom House has assessed the condition of political rights and civil liberties around the world. It is used on a regular basis by policymakers, journalists, academics, activists, and many others." It publishes its measures in a yearly "Freedom in the World" report which the website claims "is the most widely read and cited report of its kind, tracking global trends in political rights and civil liberties for 50 years."

Analytic Strategy

Item Normalization. Supplementary Table 2 shows that items were asked on different scales. Some items involved binary responses (e.g., whether people mention not wanting to be neighbors with someone from a specific group). Others were asked with Likert-type scåales, such as the 1 – 10 scale that people used to rate whether behaviors were morally justifiable.

Before calculating variation across these items, we sought to normalize their scale. Our primary normalization approach was to use min-max normalization, which is a common approach in data science and machine learning[3]. Given a vector **V** = [v1, v2, … vn], we can determine min_V as the minimum value in the vector and max_V as the maximum value in the vector. We can create our normalized vector, **V'** using:

$$v_i' = \frac{v_i - v_{min}}{v_{max} - v_{min}} \tag{1}$$

In other words, each element in the new vector is the result of subtracting the minimum value of the original vector from that element, then dividing by the range of the original vector (i.e., the difference between its maximum and minimum values). This will result in a variable which has a range of 0 – 1, no matter its original scale.

As an alternative to min-max normalization, we also considered a median split approach, in which responses below the scale median were transformed to 0 and responses above the scale median were transformed to 1. Muthukrishna and colleagues[4] used a similar approach to normalize WVS items before estimating cultural fixation indices to approximate cultural distance. However, we considered this approach inferior to min-max normalization for several reasons. First, this approach omitted scale responses at the midpoint of the scale, which dropped a substantial amount of data. Second, this approach does not consider nuance in participants' responding: A person who believes homosexuality is somewhat justifiable (6 / 10) will get the same value as someone who thinks homosexuality is completely justifiable (10 / 10), even though these two individuals have very different values. Nevertheless, we report how patterns of value variation and value divergence change using this median split approach in the supplemental robustness tests.

Calculating Value Divergence Across Items. After the item normalization, we took the mean values of items for each nation. This resulted in nation-level means for each item at each timepoint. As an exploratory analysis, we fit Pearson correlations between timepoint and mean for each item, which indicates how values are changing over time across all nations. For example, there is declining interest in political action over time, with more people indicating that they "would never" sign a petition (r = 0.93), join a boycott (r = 0.89), or attend a peaceful demonstration (r = 0.53). There was also a trend towards people saying they express no confidence at all in parliament (r = 0.90) but more confidence in the military (r = -0.86).

Supplementary Table 3 lists the full set of trends in global mean endorsement of items over time. Readers should take care to interpret the correlation with respect to the item's scale, since higher values are sometimes associated with more affirmative responding.

These trends are interesting. However, we believe that they hide variation across nations. For example, the normalized mean for perceived justifiability of abortion rose in Sweden from 0.49 to 0.76 from the first WVS timepoint (1981) to the final WVS timepoint (2021). But over that same period, it fell from 0.26 to 0.16 in India. This divergence in means over time is an example of what we call "Value Divergence." We assessed value divergence across items by computing the standard deviation of country means at each WVS timepoint. We then estimated the linear trend in these SD values in a mixed effects model which we summarize in the main text, and also across a set of item-level Pearson correlations. In these models, positive coefficients represent value divergence—since the SD of country means is increasing over time—whereas negative coefficients represent value convergence.

In Supplementary Table 4, we report the correlation coefficients for each item, and median correlation coefficient across items. We also independently report these coefficients in four different subsets of nations—nations which were included in at least 2 WVS waves, 3 waves, 4 waves, and 5 waves. We conducted our analyses for these different subsets of nations because it helped protect against the possibility that value divergence was simply due to changing WVS composition over time (e.g., later waves of the WVS may have simply included more diverse countries than earlier waves). We took additional steps to mitigate this possibility when we analyzed value divergence across nations.

The individual item-level correlations in this table should be interpreted with caution because they are only made up of 7 datapoints. For this reason, we do not interpret statistical significance for these individual coefficients. However, the mixed effects model and the median correlation across items is more robust and has higher statistical power. The median correlation varied 0.34 and 0.50 for our different nation subsets, consistently indicating value divergence.

Calculating Value Distinctiveness. To calculate value distinctiveness across nations, we followed several steps. First, we computed the global median score for each value at each timepoint. For example, the median score for the item "Important Child Qualities: Hard Work" was 0.55 in the third WVS wave. Next, we computed the absolute differences between each nation's mean and the global median for each item. For example, the average response to the "Hard Work" item in Albania was 0.54, yielding an absolute difference of 0.015. Finally, we aggregated across all of these absolute differences to obtain a "value distinctiveness" score for each nation. This process of computing value distinctiveness $D_{i,j}$ can be expressed as:

$$D_i = \frac{\sum_{j=1}^{40} \left| n_{i,j} - \text{median}(N_{1,j}, N_{2,j}, N_{3,j} \ldots N_{k,j}) \right|}{40} \tag{2}$$

Where $n_{i,j}$ represents the mean value *j* of a given nation *i.* We used the median to compute the global value because it avoided outlier nations from having a large impact on the value.

Computing value distinctiveness across timepoints allowed us to estimate which nations have relatively unique values at any given time. In our main text, Figure 3 summarizes each nation's value distinctiveness score in the first and last wave that it was included in the WVS. Below, Supplementary Figure 2 visualizes value distinctiveness for each nation in each WVS wave, and

Supplementary Table 5 provides coefficients indicating value distinctiveness for each nation in each WVS wave.

In addition to estimating the value distinctiveness of individual countries at specific timepoints, we also analyzed general trends in value distinctiveness over time. Our reasoning was that, if nations are diverging in their values, the average value distinctiveness coefficient would be increasing. This would indicate that nations are "spreading out" around the global medians of values. Model 1 in Table 2 summarizes the result of this analysis, and Model 2 includes the geopolitical factors that can explain cross-national differences in value distinctiveness over time.

One key limitation of this analysis is that the global midpoint is not truly "global"—it is only the average of the countries sampled by the WVS at a particular point in time. If the WVS has become systematically more diverse in its sampling, then this could artificially create value divergence via a trend in sample heterogeneity. This is why we repeated all of our analyses for subsets of countries that had participated in 2, 3, 4, and 5 WVS waves. It is also why we conducted the decade-over-decade analysis in which we replicated the finding when looking across a subset of 32 nations which provided data in the 1990s, 2000s, and 2010s—the three decades with the greatest WVS coverage. We included one extra year (2020) in the 2010s decade because it allowed us to include an extra country (Canada) in our analyses. Supplementary Table 6 summarizes value distinctiveness scores for each decade in each country that made up the 33-nation sample.

Clustering Methodology. In our final analysis, we sought to project nations onto an *n*-dimensional value space. Greater distance in this space between a pair of nations would represent a larger difference between the values of two nations. We could also test which geopolitical variables were most strongly correlated with this pairwise distance metric.

The first step in this process was to determine how many dimensions would appropriately capture sufficient variance across values. Supplementary Figure 3 illustrates the correlations between all 40 items in our dataset. There were clear covariances across sets of values, and so we reasoned that a small number of dimensions might be sufficient to explain considerable variation across values.

To determine the optimal number of dimensions, we fit a Principal Components Analysis (PCA) on the correlation matrix of values (the same matrix displayed in Supplementary Figure 4). In this analysis, PC1 explained 65.8% of variation, and PC2 explained 15.1% of variation. No other dimension explained more than 10% of variation, so we adopted a two-dimension solution. Supplementary Figure 4 displays an elbow plot of variance explained by each PC, and the PC1 and PC2 item loadings.

We then projected nations onto a two-dimensional value space using these PC item loadings multiplied by the nation's scores on each value. This generated the plots displayed in Figure 5.

Supplemental Literature Review

Our main text includes citations of several previous studies that have examined contemporary value change across cultures. However, our word limit did not permit us to comprehensively review these studies. The aim of this supplemental literature review is to survey past research more comprehensively, and to acknowledge the many innovative studies that have come before our paper.

Apart from the Kaasa & Minkov study that we discuss in the main text[5], the most relevant prior analysis to our paper was conducted by Bonikowski. Bonikowski showed that, in a sample of 19 countries between 1990 and 2000, non-economic interactions between cultures such as belonging to the same intergovernmental organizations or historically being part of the same empire predicted cultural similarity[6]. Several other studies have asked whether cultures have been diverging or converging worldwide on a particular value or dimension of values, such secularism[7], views on permissibility of partner violence[8], support for government intervention, acceptance of homosexuality[9–12], euthanasia[13], or tolerance more broadly[10]. For instance, Roberts[11] examined longitudinal global values on homosexuality from 1981 to 2012. She found evidence for a global increase in acceptance of homosexuality but highlighted that this upswing was slower or did not happen in certain nations including the majority of Muslim countries, countries in sub-Saharan Africa and the former Soviet bloc. These analyses are consistent with our claim that opinions about the justifiability of homosexuality have diverged over time.

Other researchers have examined cultural divergence on a wide range of values but in specific populations (e.g., managers[14]) or on nation-level metrics not directly related to values, including income[15–17], life expectancy[18], maternal and child mortality[19], homicide rates[20], health expenditure[21], education systems[22], and consumer behavior patterns[23,24]. A comprehensive study by Berry and colleagues[25] tested cross-cultural convergence vs. divergence from 1960 to 2009 on an index composed of five dimensions: economic (e.g., GDP per capita), financial (e.g., domestic credit to the private sector), demographic (e.g., life expectancy), knowledge (e.g., number of patents per capita), and political (e.g., government consumption). The cultural dimension was deliberately excluded, as the first wave of the WVS took place 20 years after 1960. Their results supported global divergence but suggest that more local ties, such as membership in the same trading block, might often lead to convergence within the block and divergence across blocks. The findings regarding local convergence are consistent with work in sociology, economics, and political science that argues that a general trend toward regionalism is taking place on several geopolitical dimensions[26–28]. Our research shows that a similar trend may be characterizing contemporary changes in values.

There is some evidence from “value regionalism” in previous studies that identify convergence of specific values in specific world regions[29–33]. For example, Akailyski demonstrated that member states of the European Union and accession candidate states have converged in values from 1992 to 2011[34]. The longer a country is a member of the EU, the more its values resemble those of the EU founding states: Belgium, France, Germany, Italy, Luxembourg and the Netherlands. While other research has found that new member states (NMS) see their poorest citizens’ incomes get closer to the EU-wide median upon joining the EU[35], Akailynski’s[34] findings regarding the convergence of EU member states are separate from changes in economic development.

Further work by Akailyski & Welzel[31] found that members of the EU have been diverging from members of the Eurasian Union (Armenia, Belarus, Kazakhstan, Kyrgyzstan, and Russia), particularly with regards to emancipative values, echoing the earlier results by Berry and colleagues[25]. The authors explain the divergence between the two unions by the fact that leaders of nations whose historically predominant religions are Islam (Kazakhstan, Kyrgyzstan) or Orthodox Christianity (Armenia, Belarus, Russia) have deliberately pushed back against emancipative values (considered quintessentially “Western”) to promote their countries’ non-Western identities. Deutsch & Welzel[2] similarly cite the five nations that compose the Eurasian Union–together with Azerbaijan, Nigeria, and Turkey–as examples of countries that have lower levels of emancipative values than expected by their levels of exposure to global culture

(measured by the score on the KOF index of globalization,). The authors argue that whether emancipative values increase over time depends on whether a country is a democracy.

Several studies have focused particularly on how values have changed in East Asia. Inglehart & Welzel[22,29,30,36] maintained that modernization engenders self-expression values, which necessarily precede the rise of effective democracies. Bomhoff & Gu[29] showed that this link did not apply to wealthy and democratic East Asian societies–Japan, Taiwan, and South Korea–where values remain socially conservative, concluding that "the path to democracy is rich in variety and possibility" and that "it should not be signposted exclusively with the current Western interpretation of tolerance and self-expression" (p. 375). Akaliyski[30] expanded on this work by showing which value domains follow the developmental path suggested by modernization theorists (personal autonomy, secular identity) and which do not (individual freedom, gender equality, political liberalism, and ethnic tolerance).

Additional Analyses

Replicating Results Without Demographic Weighting. In our main analyses, we computed country means using case-weighted provided by the WVS. In other words, demographically underrepresented participants' responses were weighted more than demographically overrepresented participants' responses in an effort to make country means representative.

We also replicated our analyses without using demographic weights, and found that our results were highly consistent. A mixed effects model with observations of cross-nation value variation nested in items found that timepoint has been significantly associated with greater value variation, b = 0.004, SE = 0.0007, t(239) = 5.50, p < 0.001, β = 0.18, *95% CI*s [0.003, 0.006]. As in our main text, we also correlated timepoint with value variation for each of the 40 individual values and then analyzed the correlation coefficients of these correlations. Of the 40 values, 27 have diverged over time, with a significantly positive median correlation of 0.30 between timepoint and value variation, t(39) = 3.55, p = 0.001, M_{diff} = 0.30, 95*% CI*s [0.13, 0.48].

Replicating Results with Median Split Item Normalization. In addition to using min-max normalization, we also used a median split approach where values below the median were coded as 0, values above the median were coded as 1, and values at the median were dropped. Results were highly similar with this approach. A mixed effects model with observations of cross-nation value variation nested in items found that timepoint has been significantly associated with greater value variation, b = 0.004, SE = 0.0008, t(239) = 4.72, p < 0.001, β = 0.14, *95% CI*s [0.002, 0.005]. As in our main text, we also correlated timepoint with value variation for each of the 40 individual values and then analyzed the correlation coefficients of these correlations. Of the 40 values, 26 have diverged over time, with a significantly positive median correlation of 0.23 between timepoint and value variation, t(39) = 2.75, p = 0.009, M_{diff} = 0.23, *95% CI*s [0.06, 0.40]. Coefficients associated with each item are displayed in Supplementary Figure 5.

We were also curious about whether the same items would show high divergence scores with median split normalization, because many of the "justifiable" items used the most scale points (1 – 10), which we reasoned may have made them more sensitive to divergence. This could hypothetically have artificially inflated the association between emancipative values and value divergence. However, this did not seem to be the case. When we used median split normalization, item divergence scores remained highly correlated with loadings on the emancipative values index, r(38) = 0.47, p = 0.002, but not the secular index, r(38) = 0.05, p = 0.738. This suggests that min-max normalization did not inflate the divergence scores of items

with particular scales, and that the item-level association between emancipative relevance and value divergence was meaningful.

Replicating Results Controlling for Geographic Distinctiveness. One of the most significant challenges for our analysis is controlling for the changing composition of the WVS. Since the WVS uses distinct countries at each timepoint, it is important to determine whether longitudinal effects are real, or whether they are statistical artifacts associated with the cohort of countries changing over time. This problem is particularly pernicious because the first WVS wave included the smallest sample of countries, and these countries tended to be wealthier and more homogeneous than one would expect from a truly random cross-cultural sample.

In our main text and supplementary materials, we summarize several measures of dealing with this problem, including (a) restricting our sample to include only countries that participated in many WVS waves, (b) replicating our key analyses at the decade level across countries which provided data in the 1990s, the 2000s, and the 2010s, and (c) separating cohort effects and longitudinal effects using centering procedures. We also control for Galton's problem—the non-independence of nations—using continent random effects in our regression models. This is one parsimonious, albeit imperfect, way to control for non-independence because countries from the same continent are usually more similar than countries from different continents[37]. The method is imperfect because it does not account for interdependencies within continent. For example, India, Japan, and China are all Asian countries, but China and Japan are more similar than either country is to India.

Here we summarize one additional step that we took to controlling for the changing sample characteristics of the WVS across waves: This step involved computing the "geographical distinctiveness" of each country at each WVS wave using the same method that we computed value distinctiveness. In other words, we computed the average latitudinal and longitudinal coordinate across all countries in each WVS wave, and then we computed each country's distance from this average coordinate. For example, if Wave 1 contained mostly Western countries, then the "average" geographic coordinates would be in the West, and most countries would have low geographical distinctiveness scores because they would be relatively close to these coordinates.

This measure has two advantages. First, it allows us to estimate whether the WVS is becoming more geographically diverse over time. In regression models, we find that this measure of geographic distinctiveness is rising over time, $b = 0.15$, $SE = 0.03$, $t(10,440) = 6.05$, $p < 0.001$, $\beta = 0.01$, *95% CI*s [0.10, 0.20]. In other words, countries in the later WVS timepoints are more geographically heterogeneous than countries in the earlier WVS timepoints. Second, we can also control for this measure of geographic distinctiveness in the critical multilevel model where value distinctiveness is regressed against timepoint (see Table 2). Controlling for this measure allows us to control for temporal changes in the heterogeneity of the WVS sample. it also controls for spatial autocorrelation in a more continuous way than continent random effects, since geographically proximal countries like Japan and China will have very similar geographic distinctiveness scores in each wave (since they will be about the same distance from the "average" latitude and longitude coordinates).

Our key value divergence effect (i.e., the relationship between time and value distinctiveness) replicates in a regression model controlling for geographical distinctiveness, $b = 0.003$, $SE = 0.0005$, $t(9,351) = 5.18$, $p < 0.001$, $\beta = 0.05$, *95% CI*s [0.002, 0.004]. In other words, changes over time in the geographic heterogeneity of countries does not account for value divergence. This analysis adds an important robustness check to our original approach (continent random

effects), and further suggests that value divergence is not an artifact of changing WVS sample composition over time.

Replicating Results with Cultural Fixation Indices. In addition to using our indices of value variation and value distinctiveness, we also found evidence for value divergence using Cultural Fixation Indices (CF_{ST}). The F_{ST} metric was first developed in genetics to measure how genotype frequencies for each subpopulation differ from expectations assuming random mating. The statistic became popular because it is easy to interpret as a measure of general ratio of between-group to total variance. An F_{ST} near 0 indicates that individuals between populations are about as different as individuals within populations, whereas an F_{ST} near 1 indicates that all variance between individuals exists between populations.

In an influential 2020 paper, Muthukrishna and colleagues[4] developed a CF_{ST} metric which they applied to the items of the WVS to quantify cultural distance between populations. They published metrics of cultural distance in this paper, but also equations that researchers could use to compute cultural distance across subsets of categorical and continuous items, or even for new datasets. We translated their equations into *R* code, and computed CF_{ST} matrices for each WVS wave using the same sampling and normalizing criteria that we used for our main analyses. In particular, we computed cultural distance using the 40 items displayed in Figure 1, among all countries included in at least two waves of the WVS. Our R code for computing cultural distance is publicly available at https://osf.io/f9bz7/. In our approach, binary items or 3-level items were treated as categorical and CF_{ST} was computed using the categorical equations, whereas items with 4 or more levels were treated as continuous and CF_{ST} was computed using the continuous equations. The estimates of these analyses were sensible. For example, the lowest CF_{ST} estimate was between New Zealand and Australia in wave 7 (CF_{ST} = 0.009), whereas the highest CF_{ST} estimates was between Sweden and Bangladesh in wave 4 (CF_{ST} = 0.52). The highest CF_{ST} score featuring two countries from the same continent was between Japan and Iraq (CF_{ST} = 0.50).

After computing these CF_{ST} scores, we then estimated whether the mean cultural distance between countries has increased over time, which would be supportive of value divergence. We conducted this analysis by melting the CF_{ST} matrix from each wave into a long dataframe of pairwise country comparisons, and then binding together the wave-specific dataframes by row. Next, we fit a cross-classified model with observations nested in the first and second countries in the pairwise comparison. In this model, CF_{ST} value was regressed on timepoint. The fixed effect of timepoint was significant and positive, b = 0.003, SE = 0.0006, t(6,118) = 5.23, p < 0.001, β = 0.07, *95% CI*s [0.002, 0.004]. The effect remained significant and positive after further nesting estimates within the continents associated with each country in the pairwise comparison, b = 0.003, SE = 0.0006, t(6,092) = 5.12, p < 0.001, β = 0.06, *95% CI*s [0.002, 0.004]. These effects were consistent with our main text findings, and offer further support for worldwide value divergence. Value divergence using this CF_{ST} approach also showed non-linear growth. When we fit a second-order polynomial model, we found a significant and positive linear effect of timepoint accompanied by a significant and negative quadratic effect. Supplementary Table 7 reports the output of this model.

In Supplementary Figure 6, we illustrate the average CF_{ST} scores of each continent—contrasted with countries from other continents—over time. This figure shows that every continent became more culturally distant from other continents from 1981 to 2022. For example, the average European country became more culturally different from the average non-European country.

Replicating Analyses of Value Distinctiveness Without GDP Per Capita. GDP per capita was the only significant fixed effect in Table 2 of our main text, whereas globalization, inequality, political rights, and distance from equator were not significantly associated with value distinctiveness. In a follow-up analysis, we tested for how results changed when we removed GDP per capita from the model. Since GDP per capita is correlated with higher levels of globalization (r = 0.65), lower levels of inequality (r = -0.29), higher levels of political rights (r = 0.49), and greater distance from the equator (r = 0.16), we reasoned that it may have accounted for the non-significant associations involving these fixed effects in our main analyses. However, this did not seem to be the case. None of the other covariates reached significance in a model which did not include GDP per capita. We report these results in Supplementary Table 8.

Separating Longitudinal and Cohort Effects with Centering. In our main text, we summarize a method of separating cohort and longitudinal effects in a mixed effects model by centering values within country means[38] and then simultaneously entering the country means and the within-country centered values into regression models. When we employ this approach in our main text with the "Timepoint" variable, we can disentangle whether the same countries are becoming more value-distinct over time (the longitudinal effect) vs. whether later waves of the WVS contain more value-distinct countries than earlier waves (the cohort effect). The standard terminology is to call the centered values "within-country effects" and the country means "between country effects," but this may be confusing because our paper also features a measure of "within-country heterogeneity." To avoid confusion, we call the centered values "longitudinal effects" and the country means "cohort effects."

In our main text, we only apply this centering approach to the "Timepoint" variable. In Supplementary Table 9, we apply this approach to all of our fixed effects. This approach largely reproduces the findings in Table 2, Model 2, except that only the longitudinal effect of GDP per capita is significant, suggesting that changes in wealth are explaining value divergence; it is not simply that later waves of the WVS include more wealthy countries than earlier waves.

Non-Linear Value Divergence. In our main text, we noted that value divergence has had a non-linear functional form. Values seem to have diverged most sharply in the 1980s and 1990s, and then diverged more gradually in the 2000s and 2010s. Readers may wonder whether this non-linear form had a single "tipping point" where value divergence slowed down, or whether this deceleration was gradual. We evaluated this possibility with a series of non-linear models. We fit these models using both of our outcome variables: value variation and value distinctiveness.

Our parameterization of these non-linear models was identical to the models we report in the main text. They were multilevel models. As in the main text, the regressions involving value distinctiveness were nested within value, country, and continent, whereas the regressions involving value variation were nested within value (because the data had already been aggregated across country). We fit five models for each outcome variable: A linear model, a second-order polynomial (quadratic) model, and spline models with discontinuities at timepoint 3, timepoint 4, and timepoint 5. Spline models are also known as piecewise polynomial regressions, and they are characterized by one or more discontinuities in a functional form. We only fit models with a single discontinuity because we only had seven total timepoints—models with multiple discontinuities are characterized by more timepoints. For each model, we extracted both AIC and BIC fit to evaluate which model provided the best fit to the data. The full range of fit coefficients are given in Supplementary Table 10.

For both value variation and value distinctiveness, the quadratic model had a better fit than either the linear or either of the spline models. We report the results of this quadratic model in the main text for value distinctiveness. Supplementary Table 11 summarizes the results of the model for both value distinctiveness and value variation. For both models, we found robust positive linear effects and negative quadratic effects. This suggests that countries' values have diverged over time, and the rate of this divergence has gradually decelerated rather than changing suddenly at a single inflection point. We also report the results of the value distinctiveness model in the main text.

Within-Country Heterogeneity Analyses. We calculated within-country heterogeneity by estimating value variation across people within countries. This followed a similar approach to calculating value variation across countries. We first normalized item responses using min-max normalization, and then we took the standard deviation of responses across participants within each country (rather than across country means).

We fit linear models to test whether within-country heterogeneity is rising within countries, which would indicate within-country value divergence. However, the evidence from these models was inconclusive. When did find evidence of within-country value divergence in a mixed effects regression where within-country heterogeneity was regressed on timepoint, with random effects for item, country, and continent, $b = 0.002$, $SE = 0.0004$, $t(9{,}741) = 4.00$, $p < 0.001$, $\beta = 0.03$, *95% CI*s [0.001, 0.002]. However, the effect did not reach significance when we allowed slopes to vary across items ($p = 0.078$). Similarly, when we aggregated across items and examined the trend of within-country heterogeneity within each nation, we found a highly diverse set of effects. Values heterogeneity has been rising in countries like Algeria ($r = 0.49$), Albania ($r = 0.40$), and Iran ($r = 0.42$), but declining in countries like Lebanon ($r = -0.51$), Ghana ($r = -0.33$), and the Netherlands ($r = -0.40$). The median within-country heterogeneity trend was close to 0 (0.02), and was not significantly different from 0, $t(75)= 0.58$, $p = 0.56$, $M_{diff} = 0.02$, *95% CI*s [-0.04, 0.07]. Because of these mixed results, we do not make any claims about whether within-country heterogeneity is rising or falling across nations over time. However, the relationship between within-country heterogeneity and value distinctiveness was more robust. In our main text, we report the association in a mixed effects model controlling for GDP per capita. We report the full set of coefficients of this model in Supplementary Table 12.

Wealth and Value Distinctiveness by Continent. Supplementary Table 13 displays the full range of coefficients associated with our analysis of GDP per capita by continent. These coefficients come from a mixed effects regression with random slopes of GDP per capita to acknowledge regional variation in the effect of wealth. The dependent variable is value distinctiveness. The interactions come from a model where the continent reference is Europe. This means that the coefficients of these interactions describe the difference of effect size between GDP per capita in Europe and GDP per capita in other continents, and the effect size of wealth is significantly smaller in Asia and Africa than in Europe.

The main effects of GDP per capita in this table represent simple effects of GDP per capita in models where we have set the reference point to different continents. We report the simple effects of Europe (our original reference point), and the continents that were significantly different from Europe (Asia and Africa). The association between value distinctiveness and wealth is robust and significant in Europe. The association is non-significant in Asia and Africa, reflecting that wealth is not significantly tied to value distinctiveness in these world regions.

In our main text, we also report power estimates for the key interactions contrasts of Europe vs. Africa (observed power = 98.00%) and Europe vs. Asia (observed power = 80.20%). Power is

difficult to parse in a mixed-effects model. For example, degrees of freedom for the residuals in a standard OLS regression is simply the difference between the sample size and the number of parameters being estimated, including the intercept. In contrast, mixed effects models introduce random effects that account for variations in the data that aren't captured by fixed effects alone. This makes the calculation of degrees of freedom more complicated. Satterthwaite's approximation is one method to estimate the degrees of freedom in mixed models, and it is the default approach in the lmer models that we fit. The Satterthwaite method calculates degrees of freedom based on the variance components of the model, essentially approximating the distribution of the test statistic by considering the ratio of variances. It provides a way to compute the appropriate degrees for F-tests in the presence of the additional complexity from the random effects. However, it means that power is often not easy to infer by eye because the degrees of freedom are not an intuitive indicator of sample size, nor is there one sample size to gauge power, since variance can be decomposed across samples of countries, continents, or variables. For these reasons, standard power calculators are often inappropriate for mixed effects models.

One approach to overcome this limitation is to use simulation. For our observed power estimates, we used “simr,” which is a package that can accept mixed effects models as arguments, and then simulate the model *n* times (*n* = 500 in our simulations) to detect the observed power of a given fixed effect given the structure of the model. This approach is considered the gold standard for simulating power in mixed effects models[39], but it is still flawed, especially when applied post-hoc because there is no guarantee that the observed features of the sample data—such as the effect size of a key fixed effect—represents the true nature of the population. This risk is not as dire in our model, because our sample of countries *does* represent a large share of all the world’s countries, but we still encourage readers to treat the exact power estimates with caution.

Correlating PCs with Dimensions of Values. In our main text, we summarize analyses in which we correlated PC1 and PC2 from our PCA with previously established dimensions of values. We chose three dimensions: Welzel’s secular and emancipative value dimensions (indexed as Y010 and Y010, respectively, in the WVS), and Inglehart’s 12-item post-materialist index (indexed as Y001 in the WVS). Since these indices are stored as variables in the longitudinal WVS file, they were easy to retrieve and to correlate with the PCs.

It is important to note that these dimensions are not independent of each other. Supplementary Table 14 shows that the dimensions are correlated quite robustly. The coefficients in this table represent standardized estimates from cross-classified multi-level models with country-wave means nested in countries and waves. Because of these covariances, we chose to model the dimensions together as fixed effects in a multiple regression so that we could estimate the distinct contribution of each dimension to explaining variance in the PCs.

Supplementary Table 15 summarizes the coefficients from models where each PC was regressed on these three value dimensions. The models were cross-classified multi-level models with country-wave means nested in countries and waves. PC1 was strongly positively linked to emancipative values and also positively linked to secular values, whereas PC2 was inversely related to secular values, but not to the other dimensions. Neither PC was reliably associated with post-materialist values above and beyond the Welzel dimensions.

Visualizing Country Clusters at Each Timepoint. In the main text, Figure 5 visualizes each nation which was included in Wave 7 within a 2-dimensional value space. In Supplementary Figure 7, we illustrate this value space for the remaining 6 waves. Countries are colored based

on their continent, and their x-y coordinates indicate their position on PC1 and PC2 of the PCA that we summarize in our extended materials and methods.

Additional Examples of Value Divergence. Supplementary Table 16 lists several items where there was notable value polarization, as well as the countries where this divergence was particularly prominent. These are examples that we have hand-picked to complement our data-driven analysis, similar to the comparison between Australia and India in our main text.

Full Statistics for Clustering Regressions. In our main text, we presented beta coefficients and confidence intervals of different geopolitical variables in a regression where distance between countries' values was regressed against distance in geopolitics. Supplementary Table 17 includes the full coefficients from these models.

Supplementary References

1. WVS Database. https://www.worldvaluessurvey.org/WVSContents.jsp.
2. Deutsch, F. & Welzel, C. The diffusion of values among democracies and autocracies. *Glob. Policy* **7**, 563–570 (2016).
3. Bishop, C. M. & Nasrabadi, N. M. *Pattern recognition and machine learning*. vol. 4 (Springer, 2006).
4. Muthukrishna, M. *et al.* Beyond Western, Educated, Industrial, Rich, and Democratic (WEIRD) psychology: Measuring and mapping scales of cultural and psychological distance. *Psychol. Sci.* **31**, 678–701 (2020).
5. Kaasa, A. & Minkov, M. Are the World's National Cultures Becoming More Similar? *J. Cross-Cult. Psychol.* **51**, 531–550 (2020).
6. Bonikowski, B. Cross-national interaction and cultural similarity: A relational analysis. *Int. J. Comp. Sociol.* **51**, 315–348 (2010).
7. Li, L. M. W. & Bond, M. H. Value change: Analyzing national change in citizen secularism across four time periods in the World Values Survey. *Soc. Sci. J.* **47**, 294–306 (2010).
8. Pierotti, R. S. Increasing rejection of intimate partner violence: Evidence of global cultural diffusion. *Am. Sociol. Rev.* **78**, 240–265 (2013).
9. Andersen, R. & Fetner, T. Economic inequality and intolerance: Attitudes toward homosexuality in 35 democracies. *Am. J. Polit. Sci.* **52**, 942–958 (2008).
10. Hadler, M. & Symons, J. World society divided: divergent trends in state responses to sexual minorities and their reflection in public attitudes. *Soc. Forces* **96**, 1721–1756 (2018).
11. Roberts, L. L. Changing worldwide attitudes toward homosexuality: The influence of global and region-specific cultures, 1981–2012. *Soc. Sci. Res.* **80**, 114–131 (2019).
12. Andersen, R. & Curtis, J. Social class, economic inequality, and the convergence of policy preferences: Evidence from 24 modern democracies. *Can. Rev. Sociol. Can. Sociol.* **52**, 266–288 (2015).
13. Inglehart, R. C., Nash, R., Hassan, Q. N. & Schwartzbaum, J. Attitudes toward euthanasia: a longitudinal analysis of the role of economic, cultural, and health-related factors. *J. Pain Symptom Manage.* **62**, 559–569 (2021).
14. Ralston, D. A. *et al.* Stability and change in managerial work values: A longitudinal study of China, Hong Kong, and the US. *Manag. Organ. Rev.* **2**, 67–94 (2006).
15. Milanovic, B. Global income inequality in numbers: In history and now. *Glob. Policy* **4**, 198–208 (2013).
16. Tselios, V. Growth and convergence in income per capita and income inequality in the regions of the EU. *Spat. Econ. Anal.* **4**, 343–370 (2009).
17. Wade, R. Global trends in income inequality: What is happening, and should we worry? *Challenge* **54**, 54–75 (2011).
18. McMichael, A. J., McKee, M., Shkolnikov, V. & Valkonen, T. Mortality trends and setbacks: global convergence or divergence? *The Lancet* **363**, 1155–1159 (2004).
19. Goli, S. & Arokiasamy, P. Maternal and child mortality indicators across 187 countries of the world: Converging or diverging. *Glob. Public Health* **9**, 342–360 (2014).
20. LaFree, G. Evidence for elite convergence in cross-national homicide victimization trends, 1956 to 2000. *Sociol. Q.* **46**, 191–211 (2005).
21. Noy, S. New contexts, different patterns? A comparative analysis of social spending and government health expenditure in Latin America and the OECD. *Int. J. Comp. Sociol.* **52**, 215–244 (2011).
22. Green, A. Education and globalization in Europe and East Asia: convergent and divergent trends. *J. Educ. Policy* **14**, 55–71 (1999).
23. Mooij, M. D. Convergence and divergence in consumer behaviour: implications for global advertising. *Int. J. Advert.* **22**, 183–202 (2003).

24. De Mooij, M. The future is predictable for international marketers: Converging incomes lead to diverging consumer behaviour. *Int. Mark. Rev.* **17**, 103–113 (2000).
25. Berry, H., Guillén, M. F. & Hendi, A. S. Is there convergence across countries? A spatial approach. *J. Int. Bus. Stud.* **45**, 387–404 (2014).
26. Mansfield, E. D. & Solingen, E. Regionalism. *Annu. Rev. Polit. Sci.* **13**, 145–163 (2010).
27. Farrell, M., Hettne, B. & Van Langenhove, L. *Global politics of regionalism: theory and practice*. (JSTOR, 2005).
28. Pomfret, R. Is regionalism an increasing feature of the world economy? *World Econ.* **30**, 923–947 (2007).
29. Bomhoff, E. J. & Gu, M. M.-L. East Asia Remains Different: A Comment on the Index of “Self-Expression Values,” by Inglehart and Welzel. *J. Cross-Cult. Psychol.* **43**, 373–383 (2012).
30. Akaliyski, P. Distinct conceptions of freedom in East Asia and the protestant West underpin unique pathways of societal development. *J. Cross-Cult. Psychol.* **54**, 173–194 (2023).
31. Akaliyski, P. & Welzel, C. Clashing Values: Supranational Identities, Geopolitical Rivalry and Europe’s Growing Cultural Divide. *J. Cross-Cult. Psychol.* **51**, 740–762 (2020).
32. Kalogeraki, S. The divergence hypothesis in modernization theory across three European countries: The UK, Sweden and Greece. *Cult. Unbound* **1**, 161–178 (2009).
33. Minkov, M., Kaasa, A. & Welzel, C. Economic development and modernization in Africa homogenize national cultures. *J. Cross-Cult. Psychol.* **52**, 801–821 (2021).
34. Akaliyski, P. United in diversity? The convergence of cultural values among EU member states and candidates. *Eur. J. Polit. Res.* **58**, 388–411 (2019).
35. Goedemé, T. & Collado, D. The EU convergence machine at work. To the benefit of the EU’s poorest citizens? *JCMS J. Common Mark. Stud.* **54**, 1142–1158 (2016).
36. Inglehart, R. *Christian Welzel Modernization, Cultural Change, and Democracy The Human Development Sequence*. (Cambridge: Cambridge university press, 2005).
37. Claessens, S. & Atkinson, Q. The non-independence of nations and why it matters. (2022).
38. Enders, C. K. & Tofighi, D. Centering predictor variables in cross-sectional multilevel models: a new look at an old issue. *Psychol. Methods* **12**, 121 (2007).
39. Green, P. & MacLeod, C. J. SIMR: An R package for power analysis of generalized linear mixed models by simulation. *Methods Ecol. Evol.* **7**, 493–498 (2016).
