# Supplementary material for: Worldwide divergence of values
Source: Nat Commun. 2024 Apr 9;15:2650. doi: 10.1038/s41467-024-46581-5 (PMC11004123; doi:10.1038/s41467-024-46581-5)
Supplement: Supplementary file 3 — Reporting Summary [file 41467_2024_46581_MOESM3_ESM.pdf]

nature portfolio

Corresponding author(s): Joshua Conrad Jackson

Last updated by author(s): January 11, 2024

Reporting Summary

Nature Portfolio wishes to improve the reproducibility of the work that we publish. This form provides structure for consistency and transparency in reporting. For further information on Nature Portfolio policies, see our Editorial Policies and the Editorial Policy Checklist.

Statistics

For all statistical analyses, confirm that the following items are present in the figure legend, table legend, main text, or Methods section.

n/a	Confirmed	
☐	☒	The exact sample size (*n*) for each experimental group/condition, given as a discrete number and unit of measurement
☐	☒	A statement on whether measurements were taken from distinct samples or whether the same sample was measured repeatedly
☐	☒	The statistical test(s) used AND whether they are one- or two-sided<br>*Only common tests should be described solely by name; describe more complex techniques in the Methods section.*
☐	☒	A description of all covariates tested
☐	☒	A description of any assumptions or corrections, such as tests of normality and adjustment for multiple comparisons
☐	☒	A full description of the statistical parameters including central tendency (e.g. means) or other basic estimates (e.g. regression coefficient) AND variation (e.g. standard deviation) or associated estimates of uncertainty (e.g. confidence intervals)
☐	☒	For null hypothesis testing, the test statistic (e.g. *F*, *t*, *r*) with confidence intervals, effect sizes, degrees of freedom and *P* value noted<br>*Give P values as exact values whenever suitable.*
☒	☐	For Bayesian analysis, information on the choice of priors and Markov chain Monte Carlo settings
☐	☒	For hierarchical and complex designs, identification of the appropriate level for tests and full reporting of outcomes
☐	☒	Estimates of effect sizes (e.g. Cohen's *d*, Pearson's *r*), indicating how they were calculated

Our web collection on statistics for biologists contains articles on many of the points above.

Software and code

Policy information about availability of computer code

Data collection	No special software or code was used in data extraction in this study. We have uploaded our code to https://doi.org/10.17605/OSF.IO/F9BZ7.
Data analysis	All analyses were performed in R studio version 1.1.383.<br><br>We used the following packages:<br>rstudio, ggplot2, ggstance, foreign, haven, dplyr, reshape2, lme4, lmerTest, tidyr, RColorBrewer, countrycode, factoextra, cluster, phia, readr, maps, mapdata, Rtsne, sf, rnaturalearth, rnaturalearthdata, ggrepel, gridExtra, splines, tidyverse, simr, purr, broom

For manuscripts utilizing custom algorithms or software that are central to the research but not yet described in published literature, software must be made available to editors and reviewers. We strongly encourage code deposition in a community repository (e.g. GitHub). See the Nature Portfolio guidelines for submitting code & software for further information.

Data

Policy information about availability of data

All manuscripts must include a data availability statement. This statement should provide the following information, where applicable:

- Accession codes, unique identifiers, or web links for publicly available datasets
- A description of any restrictions on data availability
- For clinical datasets or third party data, please ensure that the statement adheres to our policy

All data are available on the open science framework at https://doi.org/10.17605/OSF.IO/F9BZ7. Data on values can also be publicly downloaded from the World Values Survey at https://www.worldvaluessurvey.org/. Data on GDP per capita, annual GDP per capita growth, and the Gini coefficient were retrieved from the World Bank (https://data.worldbank.org/). Data on Gini coefficients was retrieved from the World Inequality Database (https://wid.world/data/). Data on globalization was retrieved from the Swiss Economic Institute (https://www.theglobaleconomy.com/download-data.php). Data on political rights were retrieved from the Freedom House (https://www.theglobaleconomy.com/download-data.php). Data on religious distance was retrieved from the GlobalEconomy.com (https://www.theglobaleconomy.com/download-data.php).

Research involving human participants, their data, or biological material

Policy information about studies with human participants or human data. See also policy information about sex, gender (identity/presentation), and sexual orientation and race, ethnicity and racism.

Reporting on sex and gender	We are analyzing country-level datasets. Sex or gender differences are not applicable to our analyses.
Reporting on race, ethnicity, or other socially relevant groupings	We are analyzing country-level datasets. Sex or gender differences are not applicable to our analyses.
Population characteristics	See below for a description of research sample.
Recruitment	See below in the data collection section for information about recruitment.
Ethics oversight	The Social and Behavioral Sciences IRB Office at University of Chicago indicated that this analysis was not "human subjects research" and did not require IRB approval.

Note that full information on the approval of the study protocol must also be provided in the manuscript.

Field-specific reporting

Please select the one below that is the best fit for your research. If you are not sure, read the appropriate sections before making your selection.

☐ Life sciences ☒ Behavioural & social sciences ☐ Ecological, evolutionary & environmental sciences

For a reference copy of the document with all sections, see nature.com/documents/nr-reporting-summary-flat.pdf

Behavioural & social sciences study design

All studies must disclose on these points even when the disclosure is negative.

Study description	Analysis of worldwide value divergence using quantitative data from the World Values Survey
Research sample	The World Values Survey (WVS) is an international research program devoted to measuring the social, political, economic, and religious values of individuals around the world using regular surveys. We used their research sample aggregated to the country level.
Sampling strategy	We used all available data from the WVS. The WVS website contains comprehensive information about its research procedures (https://www.worldvaluessurvey.org). This includes information about translation procedures and fieldwork training. Samples are designed to be demographically representative of people age 18 and older residing within private residences in each country, regardless of their citizenship or language. The WVS employs probability sampling and stratified sampling to achieve these targets, but they also offer case weights to compensate for small deviations in the resulting sample with respect to gender-age (self reported), rural-urban, or education. We employed these case weights when they calculated our value means, but our results were substantively identical regardless of whether we use the weights. We report these alternative results later in the supplementary materials.<br><br>In addition to publishing data each wave, the WVS publishes a time-series file containing data from all waves. The WVS has not surveyed the same people over time in this file. Rather, each timepoint contains a demographically representative snapshot of people in a country at a particular point in time.<br><br>The WVS also publishes a list of variables indicating which items are asked in different waves, and a list of countries indicating which countries are surveyed in each wave. The timeseries dataset is published in many different formats. We downloaded the Rdata

format and aggregated the data to the country level for analyses.

We have complied with all terms of service associated with websites in which we collected data. The director of the Social and Behavioral Science IRB office at University of Chicago (Cheryl Danton) determined that our research does not require IRB approval, since the nature of this research—analyzing archival deidentified data—does not quality as human subjects research.

Data collection	The WVS collects data from across nations using a global network of social scientists studying changing values and their impact on social and political life. The WVS website contains comprehensive information about its research procedures (https://www.worldvaluessurvey.org). This includes information about translation procedures, and fieldwork training. The survey is delivered through face-to-face interviews at the respondent's place of residence, and responses are either transcribed on pen-and-paper questionnaires or by the CAPI (Computer-Assisted Personal Interview) software, and a minimum number of 1200 completed interviews are required per country. The WVS requires a complete explanation of proposed sampling procedures before the start of fieldwork, and the sampling plan must be approved in writing.
Timing	Data were collected between 1981 and 2021. More details are available from https://www.worldvaluessurvey.org
Data exclusions	We focused on the 76 countries where the WVS has collected data for at least two waves, a necessary condition since we are interested in change over time.<br><br>Samples are designed to be representative of people age 18 and older residing within private residences in each country, regardless of their citizenship or language. The WVS employs probability sampling and stratified sampling to achieve these targets, but they also offer case weights to compensate for small deviations in the resulting sample with respect to sex-age, rural-urban, or education. We employed these case weights when they calculated our value means, but our results were substantively identical regardless of whether we use the weights. We report these alternative results later in the supplementary materials.<br><br>One of the scales in our analysis involved the qualities that respondents felt to be important for children to learn. For these items, respondents were not allowed to select more than 5 items. However, during data processing we realized that this rule was not always followed. To keep the questionnaire format consistent across countries and waves, we excluded all respondents (n = 20,380; 5% of the total sample) who had selected more than 5 important childhood qualities. This decision did not affect our results. All results replicated with and without excluding participants who had not followed the instructions.<br><br>There are some important changes to the WVS composition over time. The first wave was the smallest, with only 11 countries. It also contains the greatest proportion of wealthy nations. This limitation is the primary reason why we replicate our results with different subsets of nations which have participated in different a larger proportion of waves, and also replicate our results with a stable sample of nations which has participated in three decades of data collection. These analyses are less sensitive to the composition of specific waves.<br><br>One key limitation of this analysis is that the global midpoint is not truly "global"—it is only the average of the countries sampled by the WVS at a particular point in time. If the WVS has become systematically more diverse in its sampling, then this could artificially create value divergence via a trend in sample heterogeneity. This is why we repeated all of our analyses for subsets of countries that had participated in 2, 3, 4, and 5 WVS waves. It is also why we conducted the decade-over-decade analysis in which we replicated the finding when looking across a subset of 32 nations which provided data in the 1990s, 2000s, and 2010s—the three decades with the greatest WVS coverage. Table S6 summarizes value distinctiveness scores for each decade in each country that made up the 32-nation sample. We found that 20 of the 32 nations had experienced an increase in value distinctiveness from the 1990s to the 2010s.
Non-participation	This study did not involve participant recruitment. It involved a secondary analysis of correlational data.
Randomization	This study did not involve randomization. It involved a secondary analysis of correlational data.

Reporting for specific materials, systems and methods

We require information from authors about some types of materials, experimental systems and methods used in many studies. Here, indicate whether each material, system or method listed is relevant to your study. If you are not sure if a list item applies to your research, read the appropriate section before selecting a response.

Materials & experimental systems

n/a	Involved in the study	
☒	☐	Antibodies
☒	☐	Eukaryotic cell lines
☒	☐	Palaeontology and archaeology
☒	☐	Animals and other organisms
☒	☐	Clinical data
☒	☐	Dual use research of concern
☒	☐	Plants

Methods

n/a	Involved in the study	
☒	☐	ChIP-seq
☒	☐	Flow cytometry
☒	☐	MRI-based neuroimaging

Plants

Seed stocks	NA
Novel plant genotypes	NA
Authentication	NA
